# Combination probes with intercalating anchors and proximal fluorophores for DNA and RNA detection

**DOI:** 10.1093/nar/gkw579

**Published:** 2016-07-01

**Authors:** Jieqiong Qiu, Adam Wilson, Afaf H. El-Sagheer, Tom Brown

**Affiliations:** 1 Department of Chemistry, University of Oxford, Chemistry Research Laboratory, 12 Mansfield Road, Oxford, OX1 3TA, UK; 2 Chemistry Branch, Department of Science and Mathematics, Faculty of Petroleum and Mining Engineering, Suez University, Suez, 43721, Egypt

## Abstract

A new class of modified oligonucleotides (combination probes) has been designed and synthesised for use in genetic analysis and RNA detection. Their chemical structure combines an intercalating anchor with a reporter fluorophore on the same thymine nucleobase. The intercalator (thiazole orange or benzothiazole orange) provides an anchor, which upon hybridisation of the probe to its target becomes fluorescent and simultaneously stabilizes the duplex. The anchor is able to communicate via FRET to a proximal reporter dye (e.g. ROX, HEX, ATTO647N, FAM) whose fluorescence signal can be monitored on a range of analytical devices. Direct excitation of the reporter dye provides an alternative signalling mechanism. In both signalling modes, fluorescence in the unhybridised probe is switched off by collisional quenching between adjacent intercalator and reporter dyes. Single nucleotide polymorphisms in DNA and RNA targets are identified by differences in the duplex melting temperature, and the use of short hybridization probes, made possible by the stabilisation provided by the intercalator, enhances mismatch discrimination. Unlike other fluorogenic probe systems, placing the fluorophore and quencher on the same nucleobase facilitates the design of short probes containing multiple modifications. The ability to detect both DNA and RNA sequences suggests applications in cellular imaging and diagnostics.

## INTRODUCTION

Recent advances in Next-Generation DNA sequencing (1) have led to a revolution in our understanding of the human genome. Leading on from this, the identification and analysis of genomic variations and epigenetic control mechanisms involving RNA (2) is becoming increasingly important in the diagnosis, prognosis and stratification of disease. To take advantage of these transformative developments, the field of clinical diagnostics requires improved methods for the analysis of genomic DNA, mRNA and non-coding RNA. Such methods should be simple, sensitive and capable of high throughput. In this context fluorescence is an invaluable tool owing to its high sensitivity, widespread availability of instrumentation and ease of use. Fluorogenic probes, when used in rapid polymerase chain reaction (PCR) or real-time polymerase chain reaction (RT-PCR) assays, provide sequence specific methods to detect a range of DNA and RNA targets, to discriminate between wild-type and single-point mutations, and to analyse SNPs. The modes of action and merits of various fluorogenic methods have been reviewed (3). In the presence of its target, a fluorogenic probe may form either a fully matched duplex or, in the event of a SNP or point mutation, a partially mismatched duplex. Differences in the physical properties of these duplexes, in particular hybridisation efficiency, form the basis of the detection system. Fluorogenic probes must display a difference (preferably an increase) in fluorescence on binding to their target; this can be enhanced either by increasing the brightness of the bound state or by switching off fluorescence in the unbound state. Molecular beacons are classic examples of fluorogenic probes: (4) the hairpin conformation of the unbound state places a fluorophore in close proximity to a quencher thereby reducing the background fluorescence; hybridization to a target DNA strand causes separation of the fluorophore and quencher, resulting in a major increase in fluorescence emission. The incorporation of multiple dye moieties onto a single hybridization probe (e.g. HyBeacons) (5) confers a number of advantages. Multiple copies of the same fluorophore increase the brightness of the probe, thereby improving the limit of detection (LOD). They also reduce the fluorescence in the unhybridized probe due to collisional quenching between dye molecules. This internal quenching eliminates the requirement for hairpin structures that are essential for molecular beacon function, but which complicate melting analysis and pose particular problems when attempting to target cellular RNA. Although multiple dye-labelled probes such as HyBeacons are used extensively in clinical and forensic applications, (6–9) one drawback is the duplex destabilisation caused by the internal fluorophores. This makes it necessary to use longer probes with consequently reduced mismatch discrimination.

A number of fluorophores have been designed specifically for DNA detection, and of special interest for bioanalytical applications are dyes whose optical properties change on binding to DNA. An example is thiazole orange (TO), a DNA intercalator that is highly fluorescent in the constrained environment between base pairs in a DNA duplex (10,11). In aqueous buffer or methanol, TO is essentially non-fluorescent; rotational freedom about its methine bridge permits a non-radiative decay pathway *via* a twisted excited-state. However, on intercalation into the DNA duplex, rotational freedom is severely restricted, resulting in a fluorescent signal that is orders of magnitude brighter (12,13). TO is an indiscriminate DNA-binder (11,14) and is therefore unsuitable for sequence-specific DNA detection. However, it can be chemically attached to oligonucleotides or to peptide nucleic acid (PNA) to produce fluorogenic probes with high sequence-specificity (light-up probes and FIT probes) (15–25). Hybridisation to target DNA leads to greatly enhanced fluorescence via intercalation of TO that has the added advantage of stabilising the duplex (16,26). This added stability is important, permitting the use of short probe sequences that require fewer base pairs for effective hybridization and are therefore more sensitive to base pair mismatches. However, light-up probes have some limitations; the fluorescence intensity of TO is strongly inversely proportional to temperature, severely restricting its use in DNA melting applications. In addition, PNA synthesis, unlike oligonucleotide synthesis, is expensive and not universally accessible, and the physical properties of PNA oligomers are sometimes unfavourable. These factors limit the applications of both light-up and FIT probes. To circumvent these problems oligonucleotide probes labelled with thiazole orange (ECHO probes) have been developed and coupled to auxiliary dyes to provide multi-colour detection of RNA (27).

**Figure 1.**
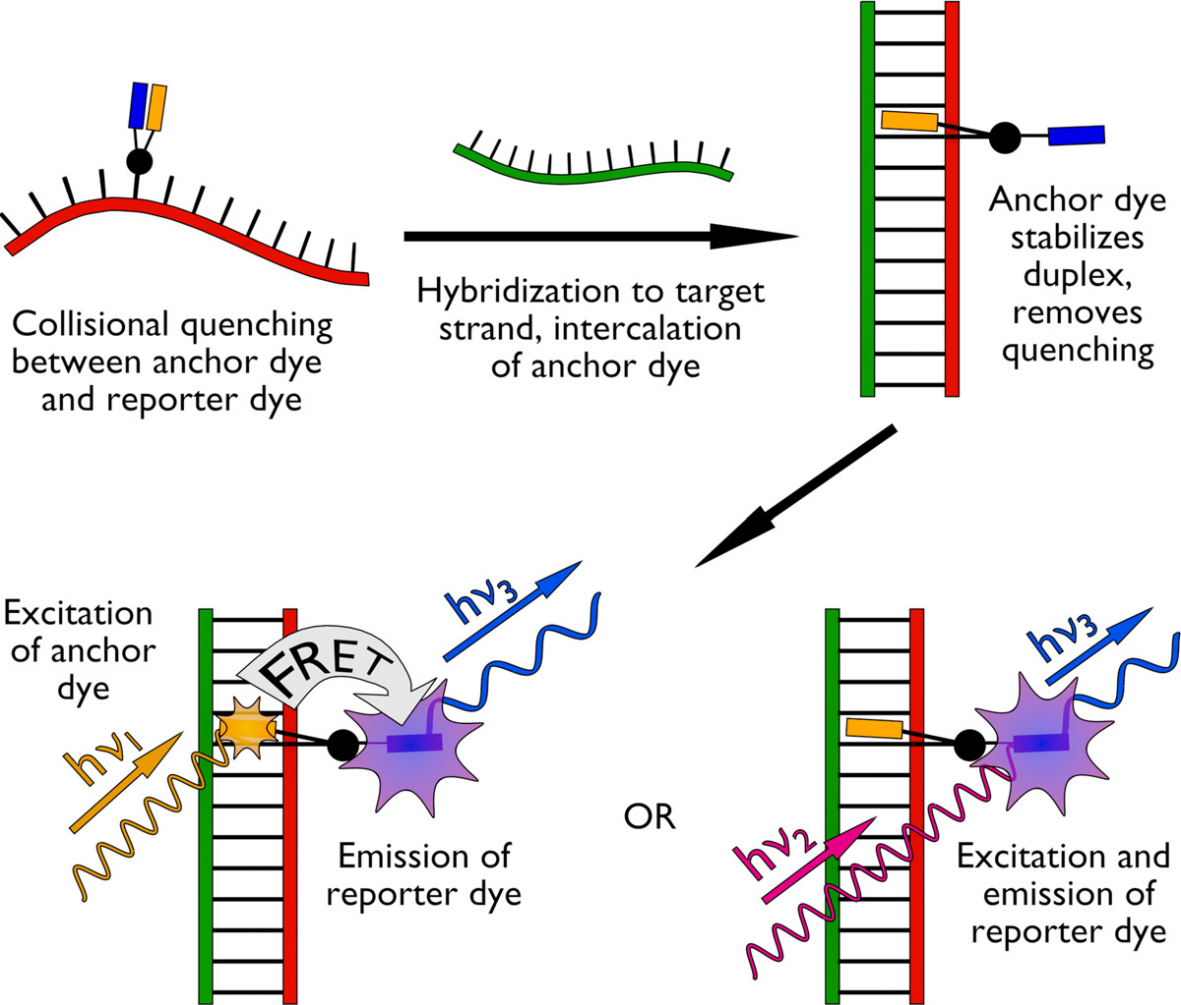
Mode of action of combination probes. The intercalative ‘anchor’ (orange) provides collisional quenching for the reporter dye (blue) in the single-stranded dark state and stabilizes the probe/target duplex upon hybridisation. The reporter dye may be interrogated via FRET from excitation of the anchor dye or by direct excitation.

It occurred to us that the most favourable features of light-up probes and HyBeacons could be combined in a single system to create novel ‘combination probes’ with improved properties (Figure 1). Inclusion of pairs of different dyes on the same nucleobase, with TO as a fluorogenic anchor and a second bright fluorophore as a reporter, would lead to efficient quenching in the unbound state only. In the duplex state, Förster resonance energy transfer (FRET) between the dyes should allow the monitoring of hybridization at different wavelengths from a single excitation source (28). Furthermore, the presence of the duplex-stabilising intercalating dye should allow the use of short probes, to provide enhanced discrimination between fully matched and mismatched complexes by melting analysis. Here we describe a number of rationally designed combination probes based on the above principles. We also demonstrate their use in fluorescence-based DNA and RNA detection and discuss future applications.

## MATERIALS AND METHODS

General methods, protocols for the synthesis of thiazole orange and benzothiazole orange phosphoramidites, oligonucleotides, UV melting analysis, asymmetric PCR using a Roche LightCycler 1.5 instrument and other biophysical methods are described in the Supplementary Methods.

### General method for solid phase labelling of oligonucleotides with fluorescent dyes by the copper(I)-catalyzed azide-alkyne cycloaddition (CuAAC) reaction

A solution of Cu^I^ click catalyst was prepared from tris (3-hydroxypropyltriazolylmethyl) amine ligand (THPTA, 2.1 μmol for one addition, 35 eq.; 4.2 μmol for two additions, 70 eq.; both in H_2_O, 5.2 µl), sodium ascorbate (3.0 μmol for one addition, 50 eq.; 6.0 μmol for two additions, 100 eq.; both in H_2_O, 3.2 μl) and CuSO_4_·5H_2_O (0.3 μmol for one addition, 5 eq., 0.6 μmol for two additions, 10 eq., both in H_2_O, 1.6 μl). The Cu^I^ solution was mixed with the fluorescent dye azide (0.6 μmol, 10 eq. for one addition; 0.9 μmol, 15 eq. for two additions) in DMSO (20 μl). The mixture was added to the TO- or BO-modified oligonucleotide on the solid support (60 nmol). All CuAAC reactions were carried out in PCR tubes (250 μl, sealed with parafilm) and heated in a heating block at 55°C for 4 h. After the click reaction, the solid support was washed with H_2_O and acetonitrile (1 ml × 3), then dried by the passage of a stream of argon gas. The resultant ligated oligonucleotide was cleaved from the solid support and deprotected by treating with concentrated aqueous ammonia solution for 4 h at room temperature in a sealed tube. All ligated oligonucleotide products were analysed by reversed-phase HPLC and characterised by mass spectrometry.

### Asymmetric PCR using BioRad CFX96 real-time PCR instrument

Reactions were undertaken using a BioRad CFX96 Real-Time PCR Instrument, with CFX Manager software (BioRad), monitoring in the following channels: FAM channel (excitation range 450–490 nm, detector range 510–530 nm), HEX channel (excitation range 515–535 nm, detector range 560–580 nm), ROX channel (excitation range 560–590 nm, detector range 610–650 nm) and Cy5 channel (excitation range 620–650 nm, detector range 675–690 nm). All PCR reactions were prepared under sterilised conditions in a LabCaire PCR workstation. Reactions were run in 0.2 ml low-profile white 8-tube strips with optically clear lids (BioRad). Sample preparation and thermal protocols for each PCR probe and polymerase are detailed in Supplementary Data.

## RESULTS AND DISCUSSION

### Selection of anchor dyes: thiazole orange (TO) and benzothiazole orange (BO)

During the development of an optimized oligonucleotide probe system that combines duplex stabilization with favourable fluorogenic properties we reasoned that use of intercalative and reporter dye combinations with different emission/excitation spectra would allow the probes to be used for multiplex applications, i.e. simultaneous analysis of several different targets. To this end we evaluated a range of ‘anchors’ that could intercalate into DNA or RNA to stabilize the probe/target hybrid. Thiazole orange (TO) and benzothiazole orange (BO) were of particular interest; they are intercalative dyes with known duplex stabilization properties. While TO has found numerous applications in DNA hybridization, BO has been used rather less often (29–31). BO has an excitation maximum at a lower wavelength than TO (444 nm as opposed to 510 nm), (32) and intercalates into DNA with a large increase in fluorescence intensity (33). Although BO forms a less stable intercalation complex than TO, the barrier to complex formation is significantly lower, presumably as a result of its smaller hydrophobic surface (32).

**Figure 2.**
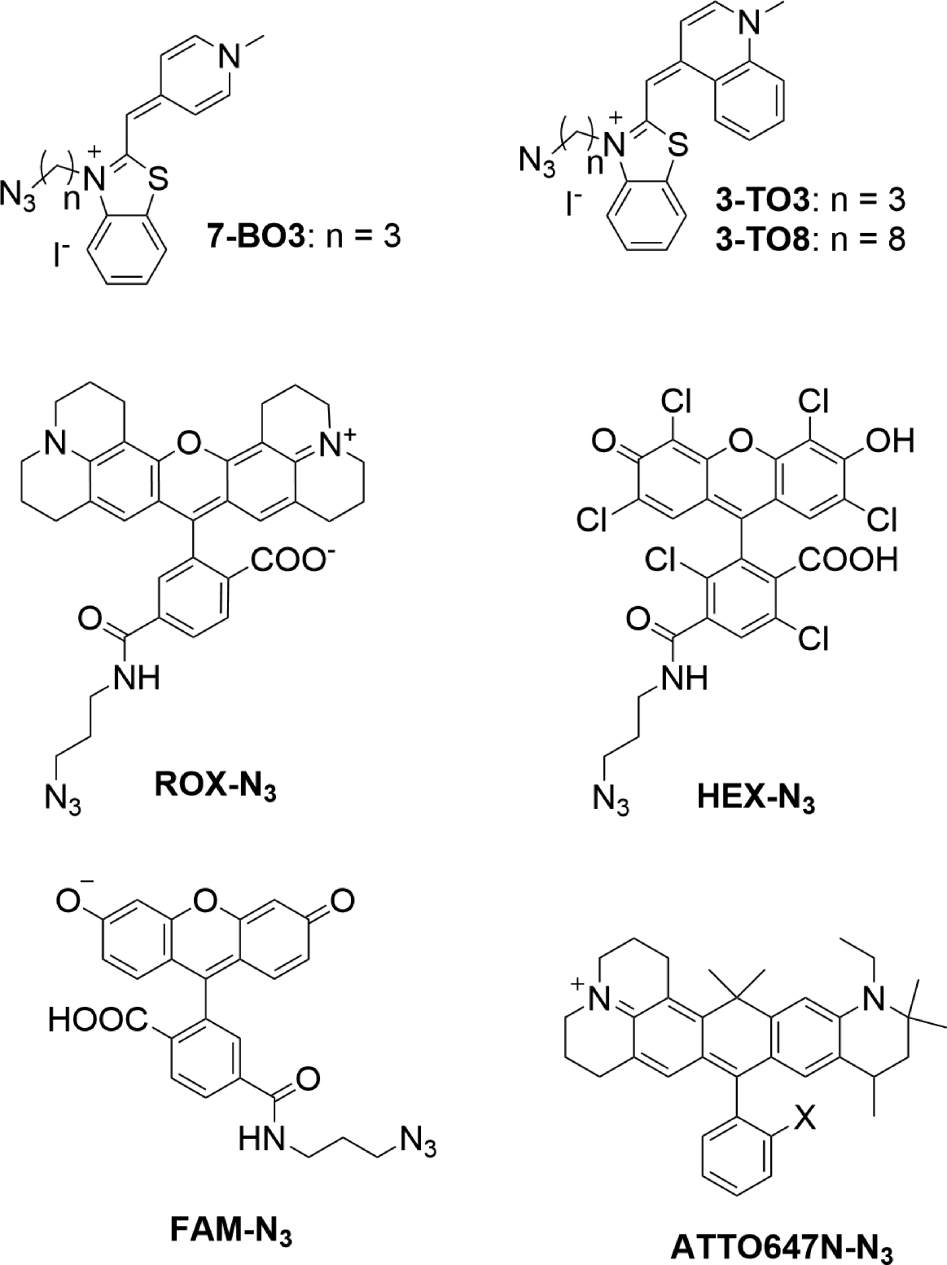
Structures of dye azides; benzothiazole orange (BO) and thiazole orange (TO) are intercalative quenchers whereas the remainder are reporter dyes. The precise structure of the ATTO647N linker has not been reported; the core chromophore is shown, X = azidoalkyl.

**Scheme 1.**
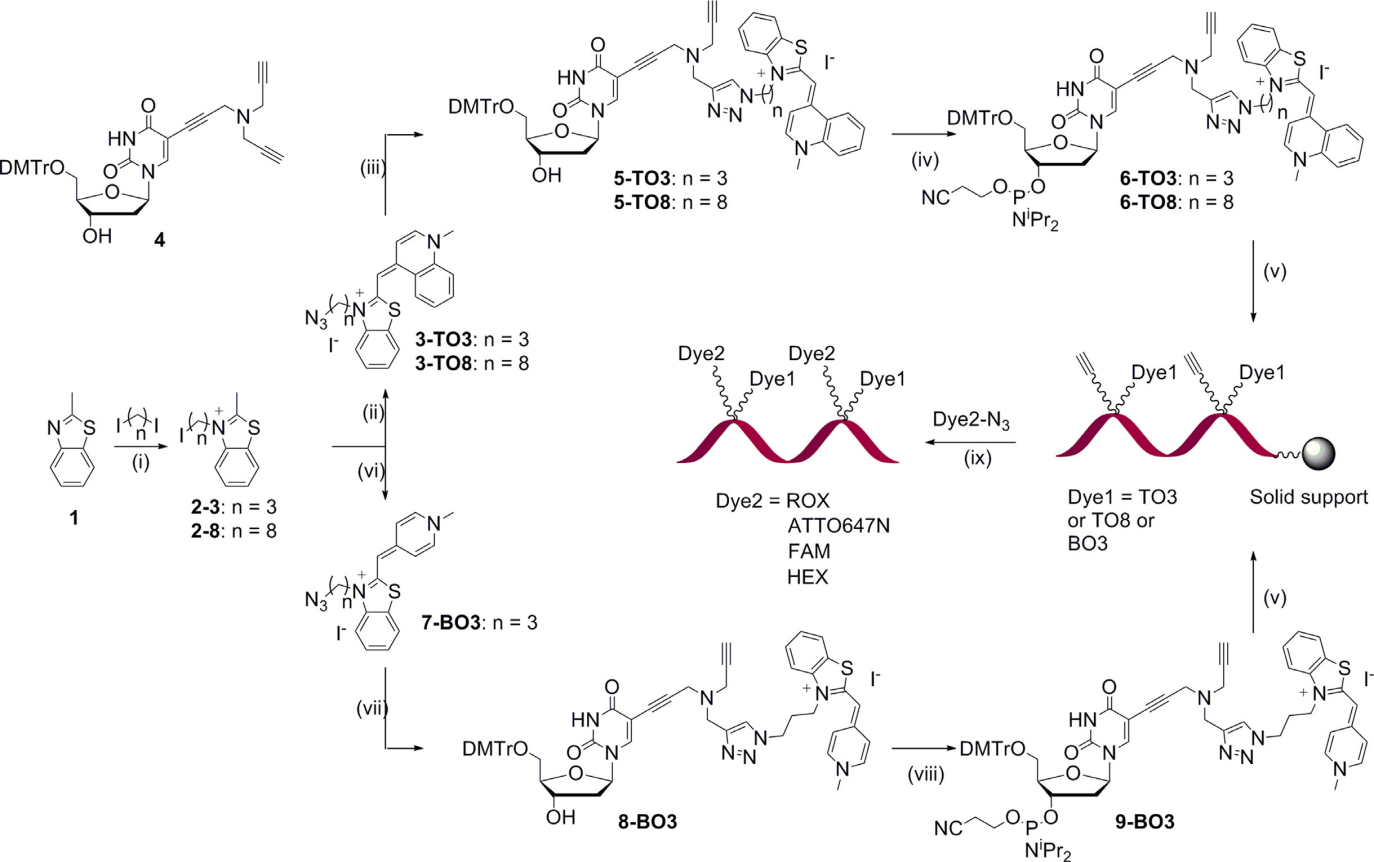
Synthesis of hybridization probes bearing an intercalating dye moiety and a free alkyne for coupling to azide-modified dyes. Conditions: (i) n = 3: MeCN, 105°C, 48 h, 64%; n = 8: MeCN, 150°C, 27 h, 67%; (ii) (a) MeCN, NaN_3_, H_2_O, RT, 19 h (n = 3, 55%; n = 8, 54%), (b) N-methylquinolinium iodide, CH_2_Cl_2_/MeOH 1:1, Et_3_N, 16 h (n = 3, 29%; n = 8, 25%); (iii) 4, DMF, H_2_O, TBTA, CuSO_4_.5H_2_O, sodium ascorbate, RT, 2 h (n = 3, 38%; n = 8, 29%); (iv) CH_2_Cl_2_, DIPEA, DMF, 2-cyanoethyl-N,N-diisopropyl chlorophosphoramidite, RT, 2 h (n = 3, 70%; n = 8, 66%); (v) solid phase oligonucleotide synthesis; (vi) (a) 4-chloro-1-methylpyridinium iodide, CH_2_Cl_2_/MeCN 1:1, Et_3_N, RT, 10 min, 10%, (b) LiN_3_, DMF, H_2_O, RT, 10 min, 76%; (vii) 4, DMF, H_2_O, TBTA, CuSO_4_.5H_2_O, sodium ascorbate, RT, 2 h, 26%; (viii) CH_2_Cl_2_, DIPEA, DMF, 2-cyanoethyl-N,N-diisopropyl chlorophosphoramidite, RT, 2 h, 64%; (ix) (a) THPTA, CuSO_4_.5H_2_O, sodium ascorbate, H_2_O, DMSO, 55°C, 4 h, (b) concentrated aqueous ammonia, RT, 4 h.

Initial work focused on the development of a strategy for the incorporation of these intercalative dyes into the synthetic probes that would be compatible with the subsequent addition of a second proximal reporter dye. We decided on a click chemistry approach, as this is known to be compatible with DNA (34). We therefore needed to prepare azide derivatives of BO and TO (Figure 2) suitable for reaction with an alkynyl group appended to the thymine nucleobase of the target monomer (Scheme 1). To prepare azide-modified TO and BO dyes, 2-methylbenzothiazole **1** was reacted with either 1,3-diiodopropane or 1,8-diiodooctane to give **2-n** (n = 3 or 8 for TO, n = 3 for BO). These two different alkyl spacers were chosen to investigate the effect of linker length on dye intercalation. Conversion of iodo-functionalized **2-n** to the corresponding azide followed by coupling to N-methylquinolinium iodide under basic conditions afforded **3-TOn**. Modified thymine nucleoside **4** (35,36) provided the starting point for a convenient DNA scaffold for single or multiple additions of dye moieties into oligonucleotides by CuAAC reactions. Stoichiometric control was used to reduce the formation of unwanted bis-clicked nucleoside products, allowing single dye-modified nucleosides **5-TOn** to be isolated in satisfactory yield. These nucleosides were then converted to the corresponding phosphoramidites **6-TOn** for incorporation into DNA probes during solid-phase oligonucleotide synthesis. A modified route was taken for the preparation of BO-containing phosphoramidites because exhaustive attempts to couple N-methylpyridinium iodide to **2-n** (n = 3) were unsuccessful. Fortunately, use of 4-chloro-1-methylpyridinium iodide, (37) followed by conversion of iodo to azide, afforded **8-BO3** in acceptable yield. The TOn (n = 3 or 8) or BO3-modified thymidine nucleotides were used in place of unmodified thymidines at one or two positions in probe sequences during automated solid-phase oligonucleotide synthesis. The resulting probes contained dye moieties and free alkyne groups, ready for direct fluorescence studies and further functionalisation with reporter dyes in subsequent click reactions. A number of reporter dye azides, selected for their bright fluorescence and compatibility with PCR applications (Figure 2, ROX, HEX, ATTO647N and FAM), were added to the same modified thymine bases as partners for TO or BO. The azide-modified dyes were allowed to react with alkyne moieties on the resin-bound probes in the presence of THPTA (tris(3-hydroxypropyltriazolylmethyl)amine), (38) sodium ascorbate and cupric sulfate under an atmosphere of dry argon. After reaction and washing with water, deprotection and cleavage from the solid support was achieved by treating oligonucleotides with concentrated aqueous ammonia for 4 h at room temperature. The resulting fully functionalized 22-mer combination probes L1 and L2 (for sequences see Table 1) were purified by reversed-phase HPLC and characterized by electrospray mass spectrometry.

**Table 1.** Sequences of oligonucleotide probes and targets: S = short (13-mer), L = long (22-mer) probes. The site of incorporation of the modified thymine base into a probe strand is denoted by a red X. P = 3′-propanol PCR blocker. WT and MT target sequences are derived from the R516G locus of the wild-type CFTR gene and the G-mutant (PCR templates are in Supplementary Table S4). The site of mutation is shown in blue. In those target strands that are longer than the probe strands, the binding region is underlined. Unless otherwise stated, oligonucleotides are DNA. For identity of X see Tables 2 and 3 and Supplementary Table S1

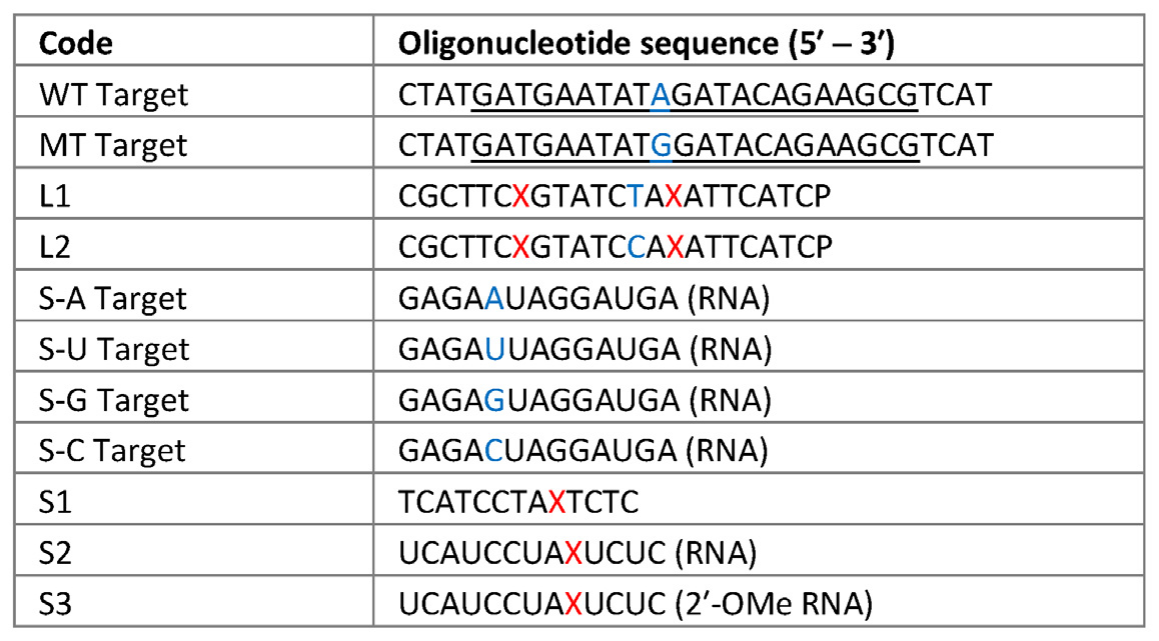	

### Effects of intercalative and Non-intercalative dyes on duplex stability

The intercalation of TO and BO into DNA duplexes increases both duplex stability and fluorescence intensity. The crowded environment between nucleobases leads to restricted rotation about the methine bridge of the dye, blocking access to non-radiative pathways to relaxation. However, despite strong fluorescence enhancement on hybridization, probes based solely on TO suffer a number of drawbacks. The fluorescence efficiency of TO has been shown to be strongly inversely temperature dependent, (11) making such probes unsuitable for stringent genetic analysis applications that utilize melting, such as SNP detection. Unfortunately, other fluorescent dyes that do not have this disadvantage are not DNA intercalators, and cause significant decreases in duplex stability. This is a drawback for fluorescent DNA probes such as HyBeacons. As discussed above, we anticipated that probes containing TO and a non-intercalative dye would combine the stabilizing effect of the TO anchor with the multi-colour capability of the non-intercalative reporter dye. We also thought that the undesirable inverse temperature dependence of TO might be less pronounced in the combination probes, particularly when the reporter dye is excited directly. With a range of biochemical applications in mind, we reasoned that such DNA probes, or their RNA or 2′-OMe RNA analogues, could satisfy these stringent criteria.

Previous studies of fluorescent hybridization probes have indicated that the stability of the probe/target duplex is strongly dependent on the length of the linker that connects the probe strand to the intercalative dye (39). Therefore, several short and long oligonucleotide probes with various combinations of 8-carbon (TO8) or 3-carbon (TO3) linkers incorporated into the sequence either singly or in pairs were hybridized to their DNA complements and duplex stability was determined by UV melting experiments (Supplementary Table S2). For all probes tested, TO3 led to a greater increase in T_m_ than TO8. This may be attributed to the higher conformational flexibility of TO8, the increased entropy of which results in inefficient base stacking and a decrease of duplex stability. For this reason, the 3-carbon linker was selected for use in combination probes. For the longer probes studied, the greatest improvement in T_m_ was achieved when two TO3-modified thymines were incorporated at different positions in the same probe sequence (**Δ**T_m_ = +8.7°C compared to the native DNA duplex). Varying the site of TO3 incorporation had little impact on duplex stability, indicating that the effect was largely sequence independent. We performed equivalent studies on BO3 incorporation which showed that BO3 is also stabilizing, though its effects are less pronounced than TO3 (**Δ**T_m_ = +3.9°C). With this knowledge in hand a number of combination probes were prepared by the methods outlined above (Tables 1 and 2). To demonstrate the diagnostic relevance of the system, the probe strands were designed to be complementary to either the wild-type cystic fibrosis transmembrane conductance regulator (CFTR) gene or the pathogenic G-mutation at the R516G locus (probe sequences L1 and L2, respectively). The CFTR gene codes for a 1480 amino acid membrane-bound glycoprotein, defects in which cause the genetic disease cystic fibrosis; mutations in the AT-rich CFTR R516G locus are of particular biomedical interest (40). Although non-intercalative dyes are known to destabilize duplexes, in the combination probes this effect was more than counteracted by the presence of the intercalative anchor dyes TO3 or BO3.

**Table 2.** Hybridization properties of anchor/reporter combination probes and non-intercalative controls: Duplex fluorescence measurements were made with the probe hybridized to its fully complementary target strand. ds/ss is the ratio of fluorescence of the double stranded probe after annealing to the fully complementary target strand (ds) to that of the single stranded probe (ss). ds/ss values were calculated from room temperature fluorescence emission scans: probes were dissolved in phosphate buffer, NaH_2_PO_4_, 10 mM, 200 mM NaCl at pH 7.4 (concentration of probe = 0.3 μM; concentration of target = 0.36 μM); T_m_ values were measured using the CFX96 Real-Time PCR instrument: for entries 1–9, KOD XL polymerase, buffer pH 7.5 was used; for entries 10–13, GoTaq polymerase, buffer pH 8.5 was used. Excitation and emission wavelengths are given in nm, with excited and emitting dyes in parenthesis (ATTO = ATTO647N). Probe strands L1 shown in red are complements of the CFTR wild-type gene; strands L2 shown in pink are complements of the G-mutant. The nucleobase opposite the site of mutation is highlighted. For melting curves see the Supplementary Data

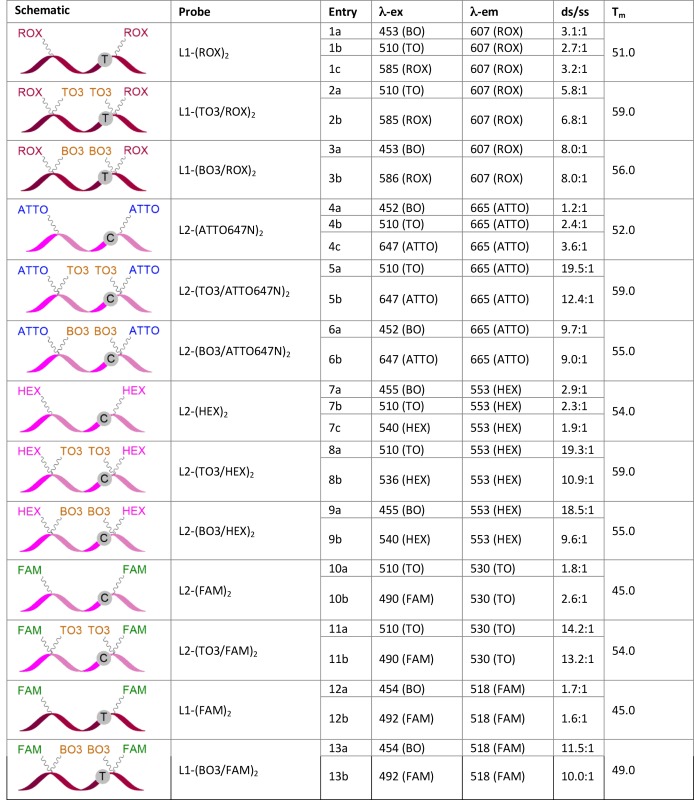						

This net stabilization was particularly pronounced when TO3 was used, with T_m_ improvements of up to +9°C relative to non-intercalative fluorescent probes (Table 2).

### Fluorescence properties of anchor–reporter combination probes

**Figure 3.**
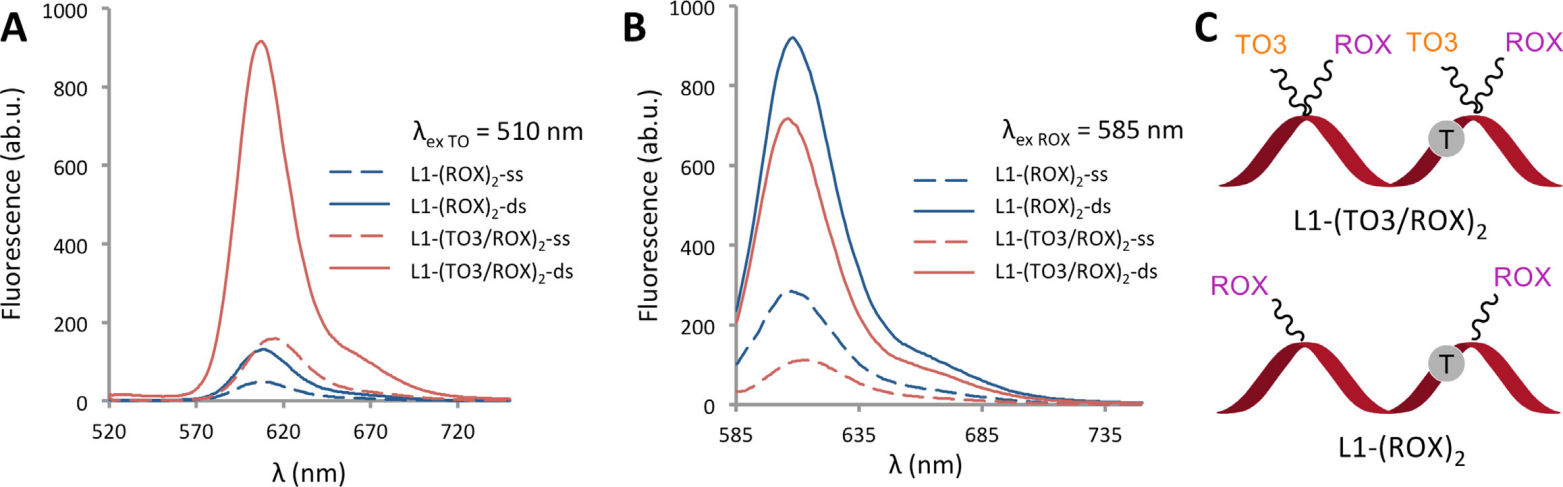
TO/ROX combination probes give a large fluorescence enhancement on hybridisation to complementary DNA. Comparison of room temperature fluorescence emission scans for the 22-mer probe L1-(TO3/ROX)_2_ and HyBeacon L1-(ROX)_2_ in phosphate buffer; NaH_2_PO_4_, 10 mM, 200 mM NaCl at pH 7.4 (concentration of probe = 0.3 μM; concentration of target = 0.36 μM). (**A**) Fluorescence emission on excitation of TO at 510 nm; (**B**) fluorescence emission on excitation of ROX at 585 nm; (**C**) schematic of the hybridization probes.

To explore the fluorescence enhancement effects of the intercalative dyes, room temperature fluorescence measurements were performed on a number of anchor–reporter combination probes both in the single stranded state (ss) and on annealing to their fully complementary target strands (ds). Values for ds/ss are in Table 2. The results were encouraging: in all cases, upon hybridization a major fluorescence enhancement relative to the corresponding non-intercalative control was achieved. This is a consequence of much higher fluorescence in the double stranded state in the FRET excitation mode (Figure 3A) and reduced background fluorescence in the ss state when the reporter dye is directly excited (Figure 3B). The low background fluorescence may be attributed to collisional quenching between proximal dye moieties.

**Figure 4.**
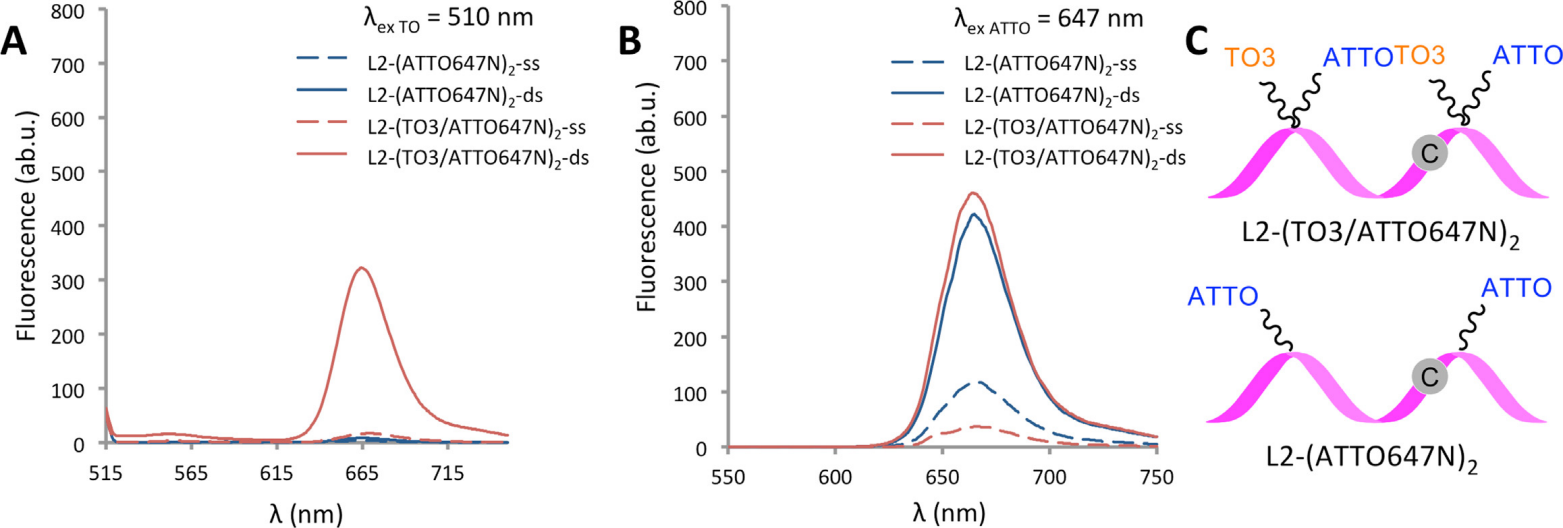
TO/ATTO combination probes give excellent fluorescence enhancement with complementary DNA. Comparison of room temperature fluorescence emission scans for the 22-mer probe L2-(TO3/ATTO647N)_2_ and HyBeacon L2-(ATTO647N)_2_ in phosphate buffer; NaH_2_PO_4_, 10 mM, 200 mM NaCl at pH 7.4 (concentration of probe = 0.3 μM; concentration of target = 0.36 μM). (**A**) Fluorescence emission on excitation of TO at 510 nm; (**B**) Fluorescence emission on excitation of ATTO647N at 647 nm; (**C**) Schematic of the hybridization probes used.

The fluorogenic properties of TO and BO were assessed for a number of anchor–reporter dye combinations. Fluorescence emission spectra of L2-(TO3/ATTO647N)_2_ were obtained on excitation of TO and ATTO647N (510 and 647 nm, respectively; Figure 4). In both cases, the emission in the ss state was low because of collisional quenching between proximal dye moieties. Hybridization to the fully complementary target strand led to intercalation of TO into the duplex, producing a reduction in quenching of ATTO647N and a very large increase in fluorescence (Table 2, entries 5a and 5b, ds/ss = 19.5:1 and 12.4:1 for FRET and direct excitation, respectively). Excitation at 510 nm resulted in an emission maximum around that of ATTO647N (665 nm) that is consistent with FRET from TO to ATTO647N. As expected, the intercalator-free probe L2-(ATTO647N)_2_ showed very little fluorescence on excitation at 452 nm (entry 4a) or 510 nm (entry 4b) whether bound or unbound, and only mild fluorescence enhancement after hybridization on excitation at 647 nm (λ_ex_ ATTO; ds/ss = 3.6:1). Similar results were obtained when TO or BO were used in conjunction with HEX or ROX; irradiation at the lower wavelength required to excite TO or BO led to FRET-induced emission of the partner dye in the DNA hybridized state.

As expected from their respective excitation and emission properties (i.e. FAM has lower excitation and emission wavelengths than TO), when FAM was used in combination with TO, FRET was observed in the reverse direction, i.e. from FAM to TO, and the resulting fluorescence was weaker than the FAM-containing HyBeacon control. However, BO, with its lower excitation wavelength, is a more suitable partner for FAM, and when BO was substituted for TO, strong fluorescence enhancement was seen on duplex formation (entry 13a, (λ_ex_ BO = 454 nm, ds/ss = 11.5:1; entry 13b, (λ_ex_ FAM = 492 nm, ds/ss = 10.0:1; Figure 5). The strong emission at 518 nm (λ_em_ FAM) on excitation at 454 nm (λ_ex_ BO) is indicative of FRET from BO to FAM; this was confirmed by the emission spectra of the control probe L1-(FAM)_2_ that lacks BO. Similar results were obtained when BO was used in conjunction with ROX or ATTO647N, with efficient FRET from BO leading to strong emission of the secondary dye (Supplementary Figure S8 and S9). The above broad spectral coverage of the dyes allowed the melting temperatures of target/probe duplexes to be monitored using a number of possible output channels of analytical devices, as discussed below. In general it should be noted that for PCR-based applications the ratio of ds/ss fluorescence is more important than overall fluorescence intensity, as sensitivity is not an issue in such applications.

**Figure 5.**
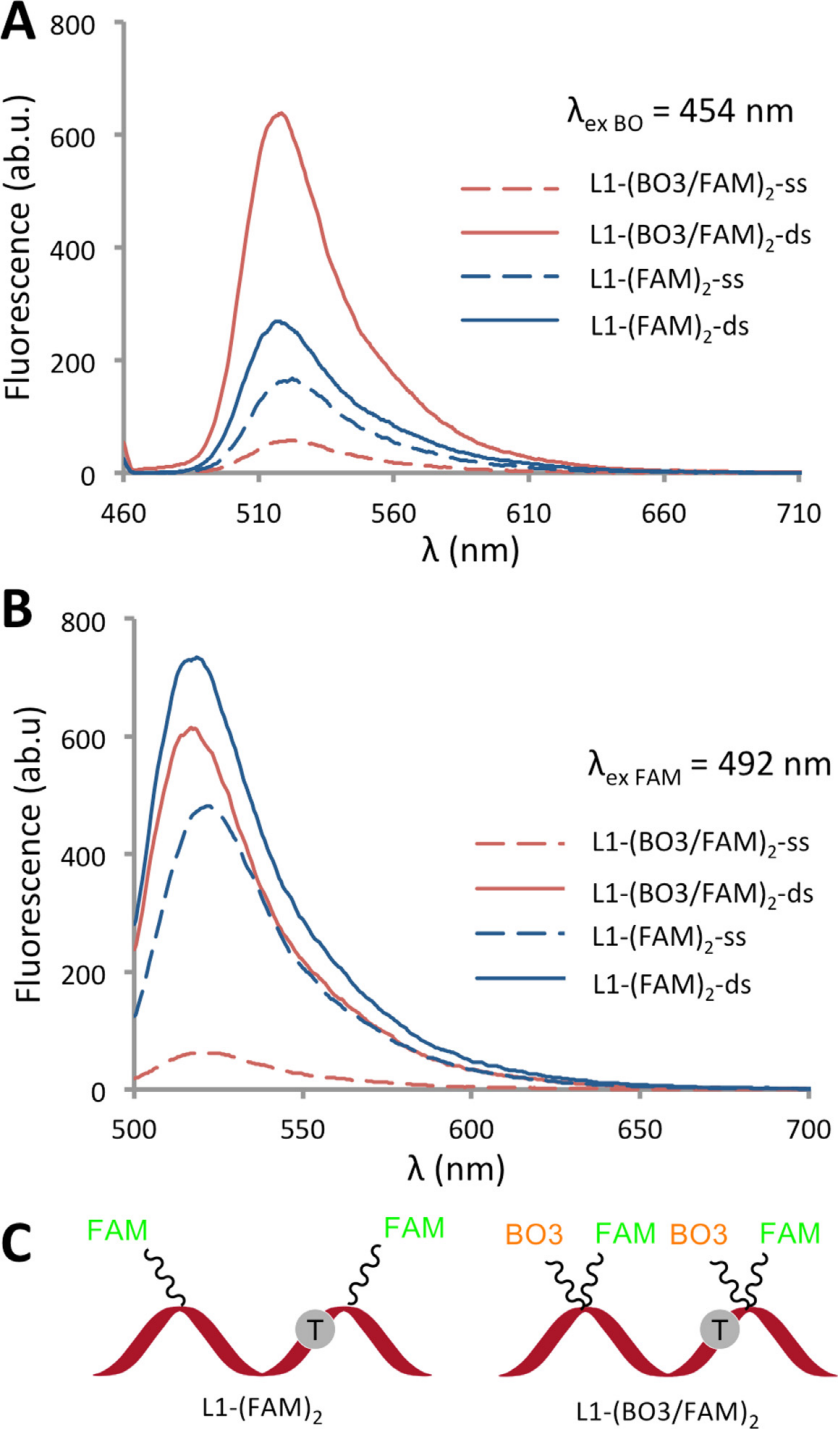
BO is a suitable partner dye for FAM in combination probes. Fluorescence emission spectra of L1-(BO3/FAM)_2_ and HyBeacon L1-(FAM)_2_ on excitation of (**A**) BO (454 nm) and (**B**) FAM (492 nm) in phosphate buffer, NaH_2_PO_4_, 10 mM, 200 mM NaCl at pH 7.4 (concentration of probe = 0.3 μM; concentration of target = 0.36 μM). (**C**) Schematic of the hybridization probes used.

### Mutation discrimination studies using anchor–reporter combination probes

Based on these encouraging biophysical results, it appeared feasible that the new anchor–reporter combination probe system could be used to detect pathogenic mutations in authentic DNA sequences. An ideal test case was the R516G locus of CFTR gene and the G-mutant, described above. In order for a hybridization probe to detect SNPs by melting temperature, there must be a significant difference in duplex stability between the fully matched and mismatched duplex. Preliminary UV-melting studies on single TO- and BO-labelled.22-mer probe strands demonstrated that differences of a single nucleobase could be readily detected by T_m_ (Supplementary Table S3). Although UV melting is an important analytical method for studying the biophysical properties of DNA analogues, fluorescence melting is the method of choice for probe-based genetic analysis applications due to its high sensitivity. Hence we studied the combination probe concept in terms of post-PCR melting on various fluorogenic PCR platforms (NB PCR platforms were chosen because it is necessary to amplify DNA targets prior to fluorogenic analysis (3)). The anchor–reporter combination probes, with their wide spectral range, were evaluated for their compatibility with the CFX96 RT-PCR instrument which has multiple excitation modes and can detect fluorescence in several output channels.

**Figure 6.**
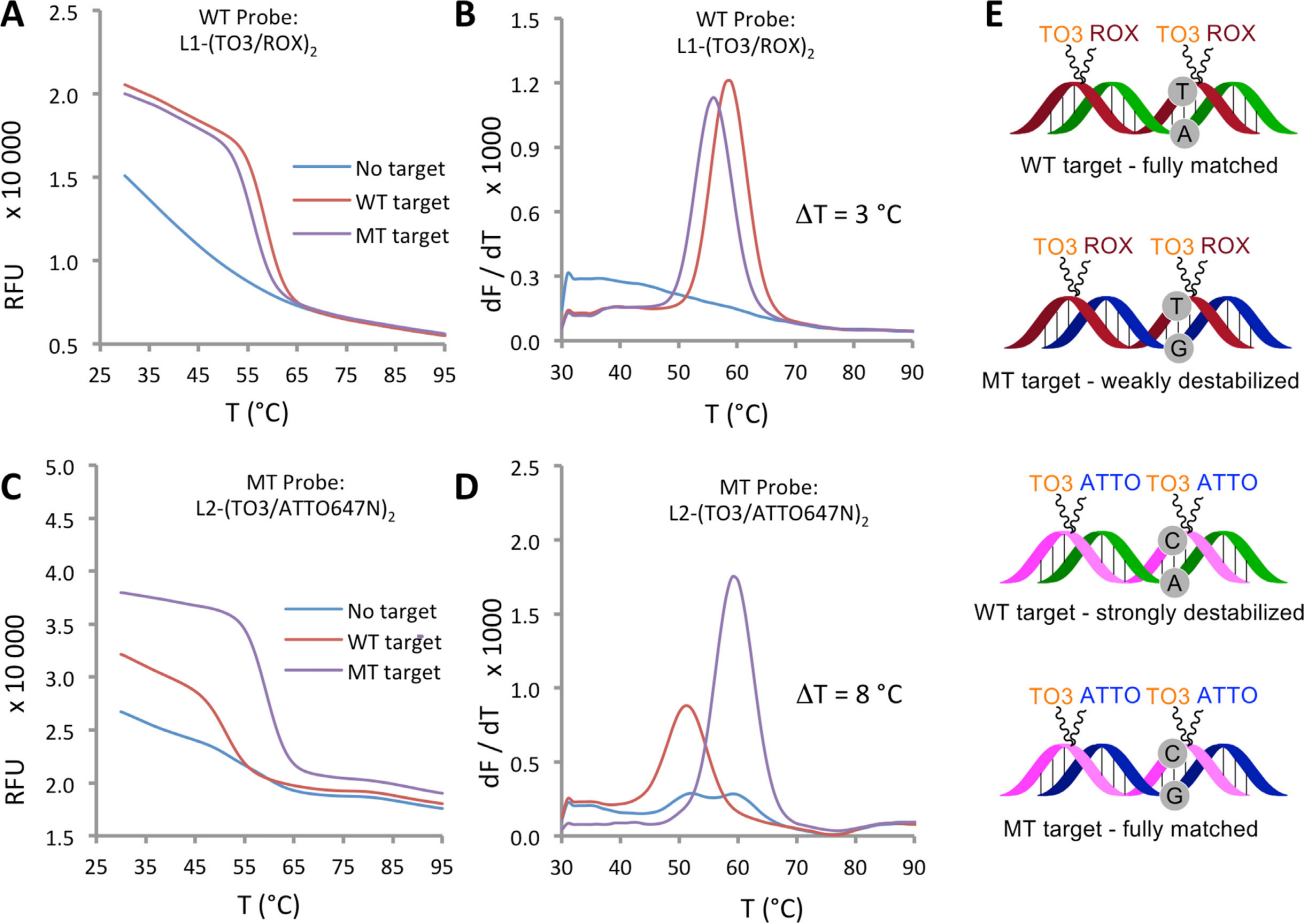
Mutation discrimination by means of base pair mismatches. Fluorescence melting curves (**A** and **C**) and derivatives (**B** and **D**) for matched and mismatched duplexes of WT probe L1-(TO3/ROX)_2_ and MT probe L2-(TO3/ATTO647N)_2_. Fluorescence was monitored in the ‘ROX’ channel (for ROX, excitation range 560–590 nm, detection range = 610–650 nm) or ‘Cy5’ channel (for ATTO647N, excitation range = 620–650 nm, detection range = 675–690 nm) of the CFX96 RT-PCR instrument. KOD XL DNA polymerase buffer (pH 7.5) was used. **Δ**T compares the mismatched and fully matched DNA duplexes. The probe/target duplexes are shown schematically (**E**) with base pairs at the site of mutation highlighted.

Probe L1-(TO3/ROX)_2_ is fully complementary to the wild-type CFTR R516G gene sequence (probes, primers and target sequences for PCR are given in the Supplementary Table S4). In fluorescence melting experiments this probe was annealed to an oligonucleotide that corresponded either to the wild-type target sequence, giving a fully-matched duplex or to the mutant sequence to give a duplex containing a single T:G mismatch. After PCR, the mutation was found to give only a modest 3°C decrease in T_m_, owing to the relatively high stability of the T:G mismatch duplex compared to the wild-type duplex that contains the least stable Watson–Crick base pair (T:A) at the same locus (Figure 6A and B). However, the combination probe L1-(TO3/ROX)_2_ has a high T_m_, (59°C) when hybridized to the wild-type target, and could clearly be shortened to enhance mismatch discrimination. Other mismatches were next investigated. Since the C:A mismatch is known to be more destabilizing than T:G, (5,41) mutant probe L2-(TO3/ATTO647N)_2_ was designed to be fully complementary to the mutant template, while forming a C:A mismatched duplex with the wild-type strand. Fluorescence melting of fully-matched and mismatched duplexes revealed an improved **Δ**T_m_ of 8°C (Figure 6C and D). These results clearly demonstrate the feasibility of SNP detection in the CFTR R516G sequence by combination probe melting curve analysis.

### Compatibility with genetic analysis instruments with a single fluorescence excitation source

**Figure 7.**
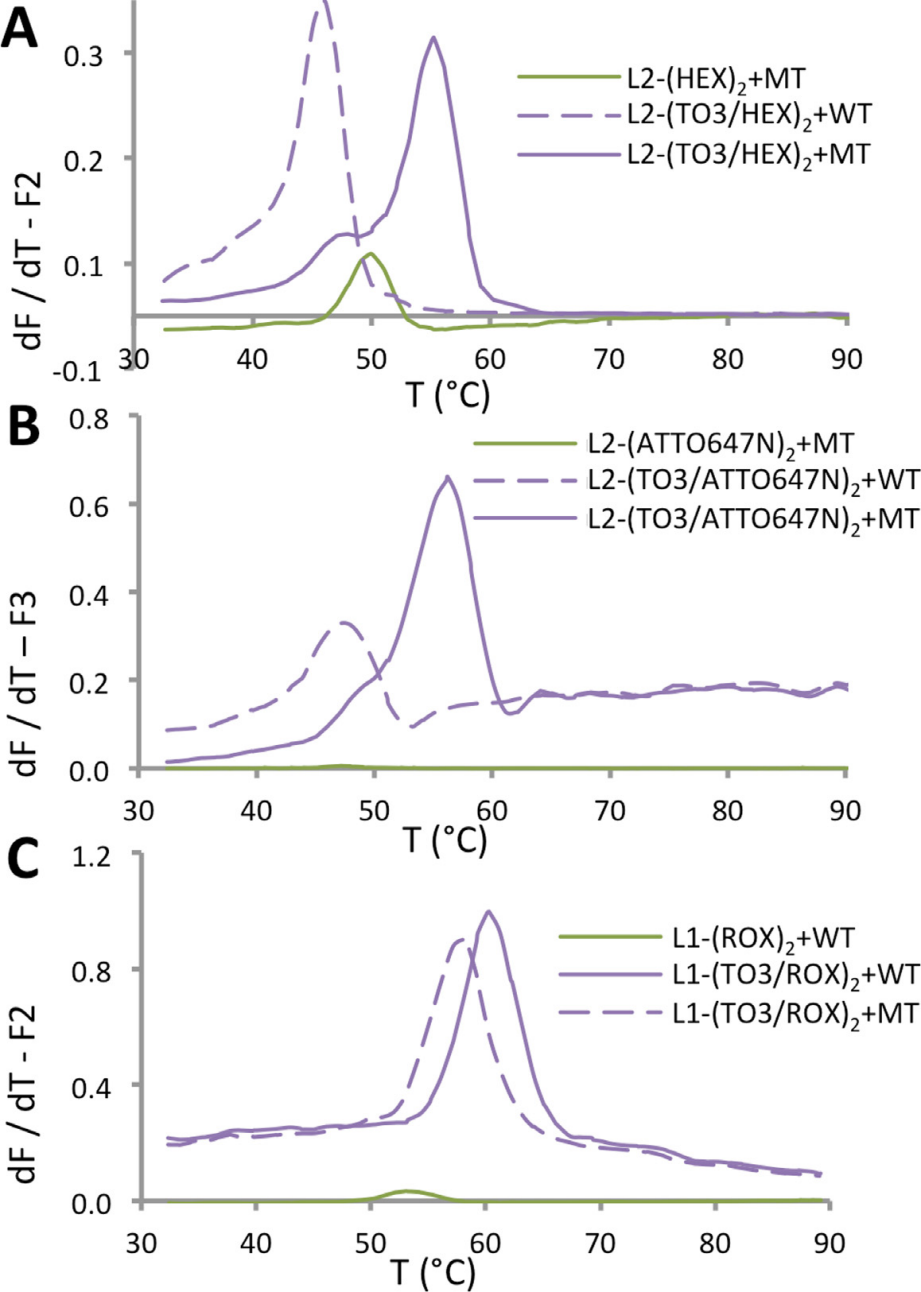
Combination probes give excellent mismatch discrimination and fluorescence enhancement with DNA targets. Fluorescence melting derivatives obtained using the Roche LightCycler for MT probes (**A**) L2-(HEX)_2_ and L2-(TO3/HEX)_2_, (**B**) L2-(ATTO647N)_2_ and L2-(TO3/ATTO647N)_2_ and (**C**) WT probes L1-(ROX)_2_ and L1-(TO3/ROX)_2_. Probes were annealed to either their fully complementary target, their (A and B) C:A mismatched WT target or (C) T:G mismatched MT target. PCR and melting experiments were performed in KOD XL buffer (pH 7.5, concentration of probe = 0.5 μM. Excitation at 488 nm; emission in the F2 channel (640 nm) for A and C, and F3 channel (705 nm) for (B).

The duplex stabilizing and fluorogenic enhancement properties of the anchor–reporter combination probes demonstrate the power of this SNP detection system. For practical applications however, the usefulness of the probes depends on their compatibility with genetic analysis instrumentation. The Roche LightCycler is a typical example of an instrument with a single excitation source (488 nm). It has three channels for recording fluorescence emission at 530 nm (F1), 640 nm (F2) and 705 nm (F3), and relies on FRET for multiplex detection applications. The device can process 32 samples in parallel. It has been used extensively in conjunction with molecular beacons, for example to determine the melting properties of intra-molecular DNA duplexes, triplexes and quadruplexes (42). The Roche LightCycler is capable of exciting the TO anchor in the present probe system, which can then communicate with different dyes via FRET to allow the emission to be recorded in the various output channels of the device. To evaluate the anchor–reporter probe system for its compatibility with the Roche LightCycler, L1-(TO3/ROX)_2_, L2-(TO3/ATTO647N)_2_ and L2-(TO3/HEX)_2_ were annealed to their complementary strands after PCR. Although the fluorescence of TO has the disadvantage of being inversely temperature dependent, importantly the fluorescence emission of the non-intercalative reporter dyes did not show such undesirable dependence. On excitation of TO at 488 nm, FRET to ROX, ATTO647N and HEX could be monitored in the F2 or F3 output channels, respectively, and the melting temperatures could be readily determined from the decrease in emission (Figure 7).

### Mutation discrimination in RNA targets using DNA, RNA and 2′-OMe RNA probes

Similar to DNA, RNA can be stabilized by intercalative dyes, although previous studies have shown that the effect is less pronounced (43,44). Single stranded RNAs are of great biological importance, not least in terms of regulation of gene expression (2). Therefore, having demonstrated the power of combination probes on DNA substrates, we determined whether the concept could be applied to RNA detection. Doing so would open up a range of biochemical applications, such as cellular imaging (45). However, the typically short lifetime of DNA probes with unmodified sugar–phosphate backbones in biological media due to enzymatic degradation severely limits their use in such applications. Hence, it is necessary to study probes based on stabilized analogues. In this context, 2′-OMe-RNA is designed to be resistant to enzymatic degradation, and probes constructed from this analogue are therefore more viable in the intracellular environment (46).

**Figure 8.**
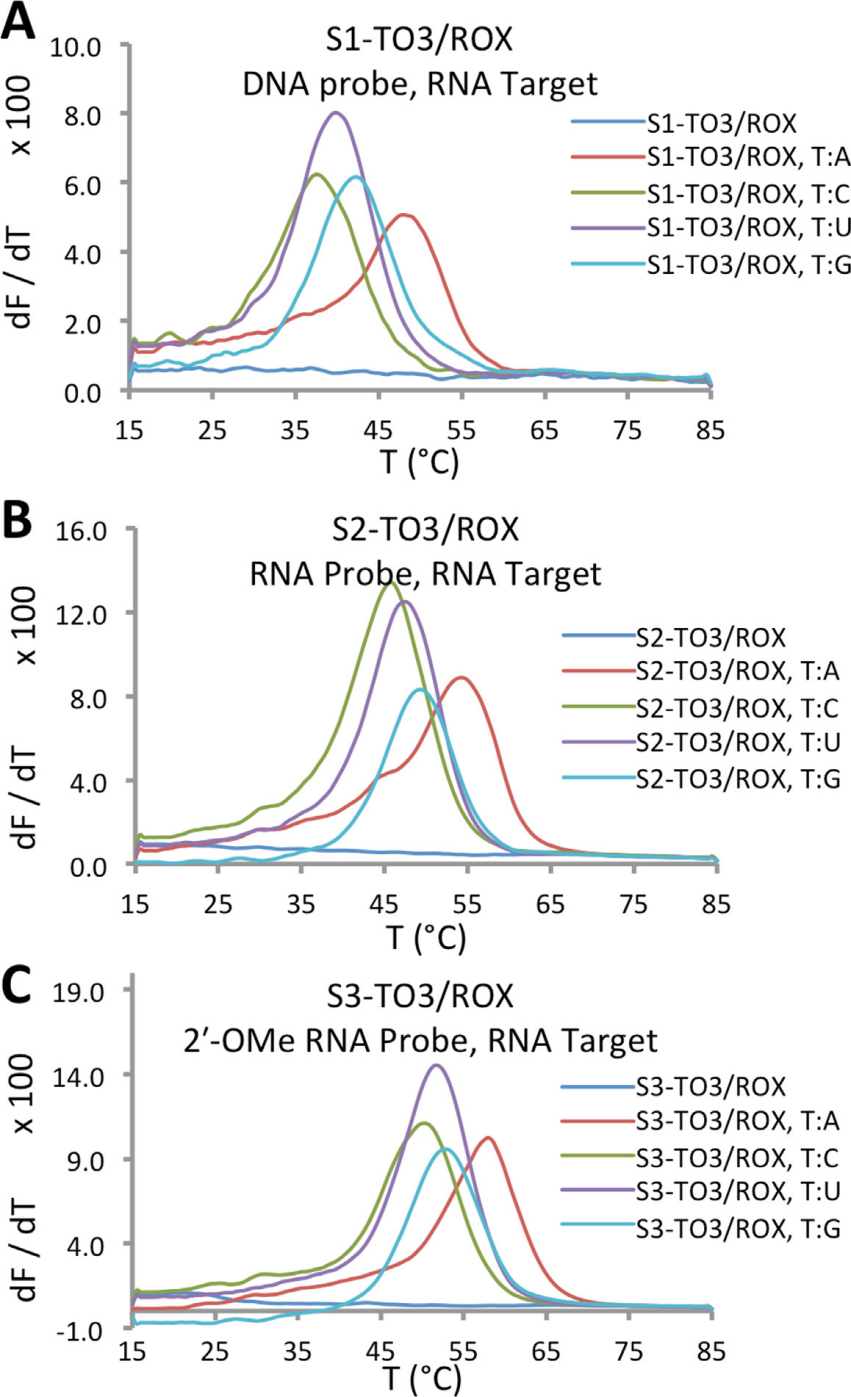
DNA, RNA and 2′-OMe protected RNA probes give excellent mismatch discrimination and fluorescence enhancement with RNA targets. Derivatives of fluorescence melting curves for RNA duplexes of (**A**) S1-TO3/ROX DNA probe, (**B**) S2-TO3/ROX RNA probe and (**C**) S3-TO3/ROX 2′-OMe RNA probe in phosphate buffer, NaH_2_PO_4_, 10 mM, 200 mM NaCl at pH 7.4 (concentration of probe = 0.5 μM; concentration of target = 0.75 μM). For controls (dark blue) no target was used. All output was monitored in the ‘ROX’ channel of the CFX96 Real-Time PCR instrument (excitation range 560–590 nm, detector range 610–650 nm).

Mutation discrimination by T_m_ is generally more effective with shorter probes. To detect single base changes in RNA target strands, we prepared doubly dye-labelled 13-mer probes S1-TO3/ROX, S2-TO3/ROX and S3-TO3/ROX, based on DNA, RNA and 2′-OMe RNA, respectively; the probes each contained a single modified thymine base bearing a TO anchor dye and a ROX FRET reporter dye (for sequences see Table 1). A number of complementary target strands differing by a single nucleotide opposite the modified thymine base in the probe strand were synthesized. The probes were annealed to their RNA targets and melting temperatures were obtained by fluorescence melting experiments, monitored in the ‘ROX’ channel of the CFX96 Real-Time PCR instrument (Figure 8). In all cases a base pair mismatch led to a readily detectable reduction in T_m_ (Table 3). Both the RNA and 2′-OMe RNA probes formed more stable duplexes with the RNA targets than their DNA analogue S1-TO3/ROX. Pleasingly, the 2′-OMe RNA probe, designed to be stable in biological media, formed a duplex with a T_m_ of 58.0°C, fully 5°C higher than the least destabilizing T:G base pair mismatch. This excellent mismatch discrimination suggests that it will be possible to differentiate between RNA targets differing by a single nucleobase under conditions compatible with live cells. We believe that the high sensitivity of these RNA probes and the mismatch discrimination made possible by the use of short probes stabilized by intercalation will be of great utility for future applications in cellular imaging and diagnostics.

**Table 3.** Mutation discrimination by T_m_ using short (S, 13-mer) DNA, RNA and 2′-OMe RNA Probes for RNA targets

Probe	Base pairing (Probe:Target)	T_m_ (°C)
DNA Probe: S1-TO3/ROX	T:A	48.5
	T:C	37.5
	T:U	40.0
	T:G	42.5
RNA Probe: S2-TO3/ROX	T:A	54.0
	T:C	46.0
	T:U	47.5
	T:G	49.5
2′-OMe RNA Probe: S3-TO3/ROX	T:A	58.0
	T:C	50.0
	T:U	51.5
	T:G	53.0

## CONCLUSION

The multi-coloured combination probes described here show promise for analytical biomedical applications against a range of DNA and RNA targets. Disease-related SNPs and point mutations in DNA can be readily detected by fluorescence melting as demonstrated using sequences corresponding to the R516G locus of the CFTR gene and its pathogenic G-mutant. Combination probes are compatible with the two common formats of genetic analytical instrumentation (FRET and non-FRET), and since bespoke specialized instrumentation is not required, the probes could be used in existing genetic testing and pathogen detection applications. Furthermore, the use of short thermodynamically and biologically stable 2′-OMe RNA probes suggests possibilities for single-molecule detection, imaging and discrimination of DNA and RNA sequences in fixed and live cells, as recently demonstrated using ECHO probes (47). The design of combination probes for a given target is simple; they do not possess inherent secondary structure and the dye combinations can be varied to allow selection of the best possible anchor–reporter pair for a given application. This is important for simultaneous detection of multiple targets. Moreover, as both fluorophore and quencher reside on the same nucleobase, designing short probes containing multiple fluorogenic modifications is a simple matter.

## Supplementary Material

SUPPLEMENTARY DATA
