# Supplementary material for: Combination probes with intercalating anchors and proximal fluorophores for DNA and RNA detection
Source: Nucleic Acids Res. 2016 Jul 1;44(17):e138. doi: 10.1093/nar/gkw579 (PMC5041472; doi:10.1093/nar/gkw579)
Supplement: SUPPLEMENTARY DATA [file supp_44_17_e138__index.html]

Combination probes with intercalating anchors and proximal fluorophores for DNA and RNA detection — Combination probes with intercalating anchors and proximal fluorophores for DNA and RNA detection — SUPPLEMENTARY DATA 

# Combination probes with intercalating anchors and proximal fluorophores for DNA and RNA detection

## SUPPLEMENTARY DATA

- SUPPLEMENTARY DATA
