# Supplementary material for: Combination probes with intercalating anchors and proximal fluorophores for DNA and RNA detection
Source: Nucleic Acids Res. 2016 Jul 1;44(17):e138. doi: 10.1093/nar/gkw579 (PMC5041472; doi:10.1093/nar/gkw579)
Supplement: SUPPLEMENTARY DATA [file supp_gkw579_nar-01109-met-g-2016-File002.docx]

Supporting Information

**Combination Probes with Intercalating Anchors and Proximal Fluorophores for DNA and RNA Detection**

Jieqiong Qiu,^a^ Adam Wilson,^a^ Afaf H. El-Sagheer^a,b^ and Tom Brown^a*^

^a^Department of Chemistry, Chemistry Research Laboratory, University of Oxford, Oxford, OX1 3TA, United Kingdom

^b^Chemistry Branch, Department of Science and Mathematics, Faculty of Petroleum and Mining Engineering, Suez University, Suez 43721, Egypt

*corresponding author [tom.brown@chem.ox.ac.uk](mailto:tom.brown@chem.ox.ac.uk)

S2. General Methods

S3. Synthesis of Compounds

S24. Oligonucleotide Synthesis and Purification

S25. General Method for Labelling of Oligonucleotides with Dyes on the Solid Phase by the CuAAC Reaction

S26. Modified Nucleoside Structures

S27. UV Melting Analysis

S28. Single and Double Dye-Modified Hybridization Probes and

their Duplex Melting Temperatures

S29. UV Melting Analysis with Free Intercalative Dyes

S30. Oligonucleotide Sequences

S32. Room Temperature Fluorescence Analysis

### S37. Asymmetric PCR using BioRad CFX96 Real-Time PCR and Roche LightCycler

S46. References

## General methods

All reagents for chemical synthesis were purchased from Aldrich, Fluka, Alfa-Aesar or ThermoFisher and used without purification with the exception of the following solvents, which were purified by distillation: THF (over sodium wire and benzophenone), DCM, DIPEA, Et_3_N and pyridine (over potassium hydroxide). Chemical transformations were carried out under an atmosphere of argon using oven-dried glassware. Column chromatography was carried out under pressure using Fisher scientific DAVISIL 60Å (35–70 micron) silica. Thin layer chromatography (TLC) was performed using Merck Kieselgel 60 F24 silica gel plates (0.22 mm thickness, aluminium backed) and the compounds were visualized by 254/365 nm UV irradiation.

^1^H NMR spectra were measured at 400 MHz on a Bruker DPX400 (AVIIIHD 400) spectrometer, or at 500 MHz on a Bruker AVIIIHD 500 spectrometer. ^13^C NMR spectra were measured at 101 MHz on a Bruker DPX400 spectrometer. ^31^P NMR spectra were recorded on a Bruker AVIIIHD 400 spectrometer at 162 or 202 MHz. All ^1^H spectra were internally referenced to the appropriate residual undeuterated solvent signal.([1](#_ENREF_1)) ^1^H and ^13^C assignments were also aided by DEPT, COSY (^1^H-^1^H) and HMQC (^1^H-^13^C) experiments.

Low-resolution mass spectra (LRMS) were recorded using electrospray ionisation (ESI^+^ or ESI^-^) on a Waters ZMD quadrupole mass spectrometer in HPLC grade methanol. High-resolution mass spectra (HRMS) were recorded in HPLC grade methanol using electrospray ionisation (EI) on a Bruker APEX III FT-ICR mass spectrometer.

The phosphoramidite monomers prepared during this study were polar and unsuitable for purification by column chromatography. They were therefore purified by repeated precipitation as described in the synthesis section below.

## Synthesis of Compounds

***N*-(3-Iodopropyl)-2-methylbenzothiazolium iodide**

2-Methylbenzothiazole (8.31 mL, 65.3 mmol, 1.0 eq.) and 1,3-diiodopropane (15.0 mL, 131 mmol, 2.0 eq.) were dissolved in acetonitrile (40 mL) under an atmosphere of dry argon. The reaction mixture was stirred under reflux for 48 h, cooled to room temperature and the solvent was removed under reduced pressure. The residue was collected and washed with cold Et_2_O (30 mL × 3), then dried under reduced pressure to give the crude product **2-3** as a yellow solid([2](#_ENREF_2)) (18.6 g, 41.9 mmol, 64%). The compound was used without further purification.

R_f_: 0.29 (10% MeOH/DCM)

^1^H NMR (400 MHz, DMSO-*d_6_*): δ 8.47 (1 H, d, *J* = 8.1 Hz, **H7**), 8.35 (1 H, d, *J* = 8.5 Hz, **H4**), 7.95–7.86 (1 H, m, **H5**), 7.85–7.76 (1 H, m, **H6**), 4.75 (2 H, t, *J* = 7.3 Hz, **H9**), 3.43 (2 H, t, *J* = 5.6 Hz, **H11**), 3.23 (3 H, s, **H8**), 2.45–2.30 (2 H, m, **H10**).

^13^C NMR (101 MHz, DMSO-*d_6_*): δ 178.2 (**C2**), 141.4 (**C3a**), 129.9 (**C5**), 129.6 (**C7a**), 128.4 (**C6**), 125.2 (**C7**), 117.1 (**C4**), 50.3 (**C9**), 32.0 (**C10**), 17.1 (**C8**), 2.7 (**C11**).

**LRMS** [ESI^+^, MeOH] *m/z* (%): 318.0 ([M-I]^+^, 100%).

***N*-(8-Iodooctyl)-2-methylbenzothiazolium iodide**

####

#### 2-Methylbenzothiazole (5.22 mL, 41.0 mmol, 1.0 eq.) and 1,8-diiodooctane (8.15 mL, 41.0 mmol, 1.0 eq.) were dissolved in acetonitrile (15 mL) under an atmosphere of dry argon. The reaction mixture was stirred under reflux for 27 h, cooled to room temperature and the solvent was removed under reduced pressure. The residue was collected and washed with cold Et_2_O (30 mL × 3), and dried under reduced pressure to give the crude product **2-8** as a yellow solid([3](#_ENREF_3)) (14.2 g, 27.5 mmol, 67%). The compound was used without further purification.

R_f_: 0.35 (10% MeOH/DCM)

^1^H NMR (400 MHz, DMSO-*d*_6_): δ 8.48–8.44 (1 H, m, **H7**), 8.34 (1 H, d, *J*= 8.5 Hz, **H4**), 7.92–7.86 (1 H, m, **H5**), 7.80 (1 H, t, *J* = 7.6 Hz, **H6**), 4.71 (2 H, t, *J* = 7.8 Hz, **H9**), 3.26 (2 H, t, *J* = 6.9 Hz, **H16**), 3.22 (3 H, s, **H8**), 1.84 (2 H, app. quin., *J*= 7.5 Hz, **H10**), 1.73 (2 H, app. quin., *J* = 6.9 Hz, **H15**), 1.44 (2 H, app. quin., *J* = 7.3 Hz, **H11**), 1.38–1.23 (6 H, m, **H12, H13, H14**).

^13^C NMR (101 MHz, DMSO-*d*_6_): δ 177.5 (**C2**), 141.3 (**C3a**), 129.9 (**C5**), 129.6 (**C7a**), 128.6 (**C6**), 125.2 (**C7**), 117.4 (**C4**), 49.6 (**C9**), 33.2 (**C15**), 30.2, 28.9, 28.3, 28.1 (**C14**, **C13**, **C12, C10**), 26.3 (**C11**), 17.4 (**C8**), 9.7 (**C16**).

**LRMS** [ESI^+^, MeOH] *m/z* (%): 388.0 ([M-I]^+^, 100%).

***N*-(3-Azidopropyl)-2-methylbenzothiazolium iodide**

*N*-(3-Iodopropyl)-2-methylbenzothiazolium iodide **2-3** (3.18 g, 7.15 mmol, 1.0 eq.) was suspended in acetonitrile (120 mL). A solution of sodium azide (580 mg, 8.92 mmol, 1.25 eq.) in H_2_O (8 mL) was added dropwise and the reaction mixture was stirred at room temperature for 19 h. The solvent was removed under reduced pressure and the residue was purified by column chromatography (1–10% MeOH/DCM) to give the compound **S1** as a yellow-green foam (2.18 g, 6.06 mmol, 55%).

^1^H NMR (400 MHz, DMSO-*d*_6_): δ 8.49 (1 H, d, *J* = 8.1 Hz, **H7**), 8.36 (1 H, d, *J*= 8.4 Hz, **H4**), 7.93–7.87 (1 H, m, **H5**), 7.84–7.78 (1 H, m, **H6**), 4.78 (2 H, t, *J* = 7.6 Hz, **H9**), 3.60 (2 H, t, *J*= 6.7 Hz, **H11**), 3.24 (3 H, s, **H8**), 2.14 (2 H, app. quin., *J* = 7.1 Hz, **H10**).

^13^C NMR (101 MHz, DMSO-*d*_6_): δ 178.1 (**C2**), 141.3 (**C3a**), 130.0 (**C5**), 129.6 (**C7a**), 128.5 (**C6**), 125.2 (**C7**), 117.2 (**C4**), 48.2 (**C11**), 47.3 (**C9**), 27.4 (**C10**), 17.6 (**C8**).

**LRMS** [ESI^+^, MeOH] *m/z* (%): 233.1 ([M-I]^+^, 100%).

***N*-(8-Azidooctyl)-2-methylbenzothiazolium iodide**

*N*-(8-Iodooctyl)-2-methylbenzothiazolium iodide **2-8** (7.00 g, 13.6 mmol, 1.0 eq.) was suspended in acetonitrile (300 mL) and a solution of sodium azide (1.33 g, 20.4 mmol, 1.5 eq.) in H_2_O (12 mL) was added dropwise. The reaction mixture was stirred at room temperature for 19 h, the solvent was removed under vacuum and the residue was purified by column chromatography (1–10% MeOH/DCM) to give the compound **S2** as a yellow-green foam (3.17 g, 7.37 mmol, 54%).

^1^H NMR (400 MHz, DMSO-*d*_6_): δ 8.45 (1 H, d, *J* = 8.1 Hz, **H7**), 8.34 (1 H, d, *J*= 8.4 Hz, **H4**), 7.89 (1 H, t, *J* = 7.5 Hz, **H5**), 7.80 (1 H, t, *J* = 7.6 Hz, **H6**), 4.71 (2 H, t, *J*= 7.8 Hz, **H9**), 3.31 (2 H, t, *J* = 6.9 Hz, **H16**), 3.22 (3 H, s, **H8**), 1.84 (2 H, app. quin., *J*= 7.4 Hz, **H10**), 1.52 (2 H, app. quin., *J* = 6.9 Hz, **H15**), 1.47–1.38 (2 H, m, **H11**), 1.37–1.28 (6 H, m, **H12, H13, H14**).

^13^C NMR (101 MHz, DMSO-*d*_6_): δ 177.5 (**C2**), 141.3 (**C3a**), 129.9 (**C5**), 129.6 (**C7a**), 128.8 (**C6**), 125.2 (**C7**), 117.3 (**C4**), 51.1 (**C16**), 49.7 (**C9**), 28.9, 28.6 (**C13,** **C14**), 28.6 (**C15**), 28.2 (**C10**), 26.5 (**C12**), 26.3 (**C11**), 16.8 (**C8**).

**LRMS** [ESI^+^, MeOH] *m/z* (%): 303.2 ([M-I]^+^, 100%).

***N*-(3-Azidopropyl)thiazole orange**

*N*-(3-Azidopropyl)-2-methylbenzothiazolium iodide **S1** (3.03 g, 8.40 mmol, 1.0 eq.) and *N*‑methylquinolinium iodide (2.74 g, 10.1 mmol, 1.2 eq.) were dissolved in DCM/MeOH (1:1, 50 mL) under an atmosphere of dry argon. Anhydrous Et_3_N (2.93 mL, 21.1 mmol, 2.5 eq.) was added, producing an immediate deep red color. The reaction mixture was stirred at room temperature for 16 h. The solvent was removed under vacuum and the residue was collected and washed with cold Et_2_O (50 mL × 3), then dried under reduced pressure. The crude mixture was purified by column chromatography (1–10% MeOH/DCM) to give the compound **3-TO3** as a red solid([4](#_ENREF_4)) (1.20 g, 2.39 mmol, 29%).

R_f_: 0.29 (EtOAc/MeOH/conc. aq. NH_3_ 5:1:1)

^1^H NMR (400 MHz, DMSO-*d_6_*): δ 8.77 (1 H, d, *J* = 8.4 Hz, **H5′**), 8.65 (1 H, d, *J* = 7.2 Hz, **H2′**), 8.20–7.91 (3 H, m, **H8′, H7, H7′**), 7.81–7.77 (2 H, m, **H6′**, **H4**), 7.65–7.59 (1 H, m, **H5**), 7.42 (1 H, t, *J* = 7.6 Hz, **H6**), 7.41 (1 H, d, *J* = 7.2 Hz, **H3′**), 6.95 (1 H, s, **H2a**), 4.66 (2 H, t, *J* = 7.2 Hz, **H8**), 4.19 (3 H, s, **H9′**), 3.60 (2 H, t, *J* = 6.4 Hz, **H10**), 2.07 (2 H, app. quin., *J* = 6.8 Hz, **H9**).

^13^C NMR (101 MHz, DMSO-*d_6_*): δ 159.8 (**C2**), 149.3 (**C4′**), 145.7 (**C2′**), 140.4 (**C3a**), 138.5 (**C8a′**), 133.7 (**C7′**), 128.7 (**C5**), 127.6 (**C6′**), 125.8 (**C5′**), 125.0 (**C6**), 124.6 (**C4a′**), 124.4 (**C7a**), 123.4 (**C7**), 118.9 (**C8′**), 113.2 (**C4**), 108.7 (**C3′**), 87.5 (**C2a**), 48.5 (**C10**), 43.6 (**C8**), 42.9 (**C9′**), 26.6 (**C9**).

**LRMS** [ESI^+^, MeOH] *m/z* (%): 374.1 ([M-I]^+^, 100%).

***N*-(3-Azidooctyl)thiazole orange**

*N*-(8-Azidooctyl)-2-methylbenzothiazolium iodide **S2** (3.17 g, 7.37 mmol, 1.0 eq.) and *N*‑methylquinolinium iodide (2.60 g, 9.58 mmol, 1.3 eq.) were dissolved in DCM/MeOH (1:1, 40 mL) under an atmosphere of dry argon. Anhydrous Et_3_N (2.57 mL, 18.4 mmol, 2.5 eq.) was added, producing an immediate deep red color. The reaction mixture was stirred at room temperature for 16 h then concentrated under reduced pressure. The residue was collected and washed with cold Et_2_O (50 mL × 3) and dried under reduced pressure. The crude mixture was purified by column chromatography (1–10% MeOH/DCM) to give the compound **3-TO8** as a red solid (1.06 mg, 1.86 mmol, 25%).

R_f_: 0.20 (10% MeOH/DCM)

^1^H NMR (400 MHz, DMSO-*d_6_*): δ 8.74 (1 H, d, *J* = 8.4 Hz, **H5′**), 8.62 (1 H, d, *J* = 7.2 Hz, **H2′**), 8.09–7.99 (3 H, m, **H8′, H7, H7′**), 7.82–7.75 (2 H, m, **H6′, H4**), 7.64–7.57 (1 H, m, **H5**), 7.44–7.38 (2 H, m, **H6, H3′**), 6.94 (1 H, s, **H2a**), 4.63 (2 H, t, *J* = 7.1 Hz, **H8**), 4.17 (3 H, s, **H9′**), 3.25 (2 H, t, *J* = 6.7 Hz, **H15**), 1.79 (2 H, app. quin., *J* = 7.4 Hz, **H9**), 1.53–1.40 (4 H, m, **H10, H14**), 1.39–1.31 (2 H, m, **H13**), 1.29–1.21 (4 H, m, **H11, H12**).

^13^C NMR (101 MHz, DMSO-*d_6_*): δ 159.1 (**C2**), 149.2 (**C4′**), 145.6 (**C2′**), 140.4 (**C3a**), 138.4 (**C8a′**), 133.8 (**C7′**), 128.8 (**C5**), 127.8 (**C6′**), 125.7 (**C5′**), 124.8 (**C6**), 124.5 (**C4a′**), 124.4 (**C7a**), 123.5 (**C7**), 119.0 (**C8′**), 113.5 (**C4**), 108.8 (**C3′**), 88.5 (**C2a**), 51.0 (**C15**), 46.1 (**C8**), 42.5 (**C9′**), 29.1, 28.9 , 28.6 (**C12**, **C13**, **C14**), 27.4 (**C9**), 26.5, 26.4 (**C11**, **C10**).

**LRMS** [ESI^+^, MeOH] *m/z* (%): 444.2 ([M-I]^+^, 100%).

**HRMS** [ESI^+^, MeOH] calc. for C_26_H_30_N_5_S, [M-I]^+^, 444.2216, found 444.2209.

**5-Iodo-5′-*O*-(4,4′-dimethoxytrityl)-2′-deoxyuridine**

5-Iodo-2′-deoxyuridine (6.05 g, 17.1 mmol, 1.0 eq.) was co-evaporated with anhydrous pyridine (10 mL × 3) and dissolved in anhydrous pyridine (20 mL). To this was added dropwise a solution of DMTrCl (6.93 g, 20.5 mmol, 1.2 eq.) in anhydrous pyridine (10 mL) over a period of 5 min and the reaction was stirred at room temperature for 1 h. The reaction was quenched by the addition of MeOH (30 mL) and then stirred for 5 min. The reaction volume was reduced under reduced pressure, diluted with DCM (30 mL) and washed with distilled H_2_O (30 mL × 2) and saturated NaHCO_3_ solution (30 mL × 2). The organic layer was dried over anhydrous Na_2_SO_4_ then filtered, and the solvent was removed under reduced pressure. Following purification by column chromatography (0–4% MeOH/DCM, 5% pyridine) compound **S3** was afforded as a white foam([5](#_ENREF_5)) (8.35 g, 12.7 mmol, 82%). R_f_: 0.44 (10% MeOH/DCM, 0.5% pyridine)

^1^H NMR (400 MHz, DMSO-*d*_6_): δ 8.03 (1 H, s, **H6**), 7.41 (2 H, d, *J* = 4.3 Hz, **H14**), 7.36–7.20 (6 H, m, **H9, H15**), 7.23 (1 H, t, *J* = 7.2 Hz, **H16**), 6.91 (4 H, d, *J* = 8.9 Hz, **H10**), 6.12 (1 H, t, *J*= 6.9 Hz, **H1′**), 4.22 (1 H, dt, *J* = 6.4, 3.2 Hz, **H3′**), 3.94–3.89 (1 H, m, **H4′**), 3.75 (6 H, s, **H12**), 3.21 (1 H, dd, *J* = 10.7, 4.9 Hz, **H5′a**), 3.17 (1 H, dd, *J* = 10.7, 3.1 Hz, **H5′b**), 2.24 (1 H, ddd, *J* = 13.7, 6.9, 6.4 Hz, **H2′a**), 2.22–2.15 (1 H, m, **H2′b**).

^13^C NMR (101 MHz, DMSO-*d*_6_): δ 161.1 (**C2**), 158.6 (**C4**), 150.6 (**C^Ar^**), 145.3 (**C^Ar^**), 144.7 (**C6**), 135.9 (**C^Ar^**), 130.2 (**C9**), 128.5 (**C****H^Ar^**), 128.1 (**CH^Ar^**), 127.2 (**C16**), 113.8 (**C10**), 86.3 (**C7**), 86.3 (**C4′**), 85.3 (**C1′**), 71.1 (**C3′**), 70.4 (**C5**), 64.20 (**C5′**), 55.0 (**C12**), 40.4 (**C2′**).

**LRMS** [ESI^+^, MeOH] *m/z* (%): 678.8 ([M+Na]^+^, 100%).

**5′-*O*-(4,4′-Dimethoxytrityl)-5-[(*N,N*-dipropyn-2-yl)-3-aminopropyn-1-yl)]-2′-deoxyuridine**

5-Iodo-5′-*O*-(4,4′-dimethoxytrityl)-2′-deoxyuridine **S3** (5.25 g, 8.14 mmol, 1.0 eq.) was co‑evaporated with anhydrous DMF (4 mL × 3) and dried for 2 h under reduced pressure. Compound **S3** and copper(I) iodide (0.31 g, 1.63 mmol, 0.2 eq.) were dissolved in anhydrous DMF/Et_3_N (1:1, 40 mL), followed by the addition of tripropargylamine (4.61 mL, 32.6 mmol, 4.0 eq.). The reaction was stirred under an atmosphere of dry argon for 10 min at room temperature, then tetrakis(triphenylphosphine)palladium(0) (0.94 g, 0.81 mmol, 0.1 eq.) was added. The reaction mixture was stirred at room temperature for 14 h. After the solvent was removed under vacuum, DCM was added and the mixture was washed with distilled H_2_O (50 mL × 2) and brine (50 mL × 2). The organic layer was dried over anhydrous Na_2_SO_4_ then filtered, and the mixture was concentrated under reduced pressure. Following purification by column chromatography (20–60% EtOAc/toluene, 0.5% pyridine) compound **4** was afforded as a colorless foam([6](#_ENREF_6)) (3.40 g, 5.15 mmol, 63%).

R_f_: 0.18 (50% EtOAc/toluene, 0.5% pyridine)

^1^H NMR (400 MHz, DMSO-*d*_6_): δ 11.70 (1 H, s, **NH**), 7.93 (1 H, s, **H6**), 7.41 (2 H, d, *J*= 7.6 Hz, **H14**), 7.34–7.20 (7 H, m, **H9**, **H15**, **H16**), 6.88 (4 H, d, *J* = 8.2 Hz, **H10**), 6.12 (1 H, t, *J* = 6.6 Hz, **H1′**), 5.35 (1 H, d, *J* = 4.3 Hz, **OH**), 4.32–4.25 (1 H, m, **H3′**), 3.95–3.91 (1 H, m, **H4′**), 3.73 (6 H, s, **H12**), 3.36 (2 H, s, **H19**), 3.32 (4 H, d, *J*= 2.1 Hz, **H20**), 3.28–3.24 (1 H, m, **H5a′**), 3.22 (2 H, t, *J* = 2.1 Hz, **H22**), 3.10 (1 H, dd, *J* = 10.1, 2.2 Hz, **H5b′**), 2.33–2.24 (1 H, m, **H2a′**), 2.23–2.16 (1 H, m, **H2b′**).

^13^C NMR (101 MHz, DMSO-*d*_6_): δ 162.1 (**C2**), 158.5 (**C4**), 150.0 (**C^Ar^**), 145.7 (**C^Ar^**), 143.5 (**C6**), 136.8 (**C^Ar^**), 130.2 (**C9**), 128.4 (**CH^Ar^**), 128.0 (**CH^Ar^**), 127.2 (**C16**), 113.7 (**C10**), 98.8 (**C5**), 88.1 (**C7**), 86.3 (**C4′**), 86.2 (**C17**), 85.5 (**C1′**), 79.4, 77.4 (**C18**, **C21**), 76.5 (**C22**), 70.9 (**C3′**), 64.2 (**C5′**), 55.5 (**C12**), 42.5 (**C19**), 41.5 (**C20**), 40.2 (**C2′**).

**LRMS** [ESI^+^, MeOH] *m/z* (%): 666.0 ([M+H]^+^, 100%).

**5′-*O*-(4,4′-Dimethoxytrityl)-5-[(*N,N*-dipropyn-2-yl)-3-aminopropyn-1-yl)]-2′-deoxyuridine (*N*-diisopropylamino)-2-cyanoethyl phosphoramidite**

5′-*O*-(4,4′-Dimethoxytrityl)-5-[(*N,N*-dipropyn-2-yl)-3-aminopropyn-1-yl)]-2′-deoxyuridine **4** (300 mg, 0.46 mmol, 1.0 eq.) was dissolved in anhydrous DCM (3 mL) under an atmosphere of dry argon. Anhydrous DIPEA (200 µL, 1.15 mmol, 2.5 eq.) was added, followed by addition of 2‑cyanoethyl-*N,N*-diisopropylchlorophosphoramidite (120 µL, 0.55 mmol, 1.2 eq.) dropwise. The reaction mixture was stirred at room temperature for 1.5 h and transferred under argon into a separating funnel containing degassed DCM (15 mL). The mixture was washed with degassed saturated aqueous KCl (20 mL) and the organic layer was dried over anhydrous Na_2_SO_4_, filtered, and concentrated under vacuum. The crude phosphoramidite product was purified by reverse precipitation by dissolving in anhydrous DCM (1 mL) and adding anhydrous hexane (20 mL). After repeated reverse precipitation (×3), the product **S4** was isolated as a colorless foam([6](#_ENREF_6)) (303 mg, 0.35 mmol, 77%).

R_f_: 0.65 (5% MeOH/DCM, 0.5% pyridine)

^1^H NMR (400 MHz, CD_2_Cl_2_-*d*_2_): δ 8.00 (0.5 H, s, **H6**), 7.96 (0.5 H, s, **H6**), 7.45 (2 H, d, *J*= 7.6 Hz, **H14**), 7.38–7.29 (6 H, m, **H9, H15**), 7.27–7.21 (1 H, m, **H16**), 6.87 (2 H, d, *J*= 8.7 Hz, **H10**), 6.85 (2 H, d, *J*= 8.7 Hz, **H10**), 6.29–6.22 (1 H, m, **H1′**), 4.66–4.58 (1 H, m, **H3′**), 4.24–4.15 (1 H, m, **H4′**), 3.89–3.76 (8 H, m, **H26** overlaps with s, **H12** at 3.79), 3.76–3.51 (4 H, m, **H23**, **H5′**), 3.43–3.31 (8 H, m, **H19**, **H20**, **H22**), 2.63 (1H, t, *J* = 6.2 Hz, **H24a**), 2.60–2.51 (1 H, m, **H2′a**), 2.47 (1 H, t, *J* = 6.2 Hz, **H24b**), 2.35–2.29 (1 H, m, **H2′b**), 1.20–1.15 (9 H, m, **H27**), 1.10 (3 H, d, *J* = 6.7 Hz, **H27**).

^31^P NMR (162 MHz, CD_2_Cl_2_-*d*_2_): δ 149.81, 148.74.

**5´-DMT Thiazole Orange C3 dU**

*N*-(3-Azidopropyl)-2-[(1,4-dihydro-1-methylquinolin-4‑ylidene)methyl]benzothiazolium iodide **3-TO3** (400 mg, 0.80 mmol, 1.0 eq.) and 5′-*O*-(4,4′-dimethoxytrityl)-5-[(*N,N*-dipropyn-2-yl)-3-aminopropyn-1-yl)]-2′-deoxyuridine **4** (633 mg, 0.96 mmol, 1.2 eq.) were dissolved in DMF (8 mL), to which was added tris(benzyltriazolylmethyl)amine TBTA (212 mg, 0.40 mmol, 0.5 eq.), and sodium ascorbate (317 mg, 1.60 mmol, 2 eq.) in water (1 mL), followed by a solution of copper(II) sulfate (100 mg, 0.40 mmol, 0.5 eq.) in water (1 mL). The reaction mixture was stirred at room temperature for 2 h, then the solvent was removed under reduced pressure, and the residue was dissolved in 10% MeOH in DCM (25 mL). The mixture was washed with a 5% Na_2_EDTA solution (25 mL × 3), then with distilled H_2_O (30 mL × 2) and brine (30 mL × 2). The organic layer was dried over anhydrous Na_2_SO_4_ then filtered, and the solvent was concentrated under reduced pressure. Following purification by column chromatography (1–10% MeOH/DCM, 0.5% pyridine) compound **5‑TO3** was afforded as an orange foam (350 mg, 0.30 mmol, 38%).

R_f_: 0.17 (EtOAc/MeOH/conc. aq. NH_3_ 5:1:1)

^1^H NMR (400 MHz, DMSO-*d*_6_): δ 11.68 (1 H, s, **NH**), 8.74 (1 H, d, *J* = 8.5 Hz, **H41**), 8.64 (1 H, d, *J* = 7.2 Hz, **H37**), 8.09–7.97 (4 H, m, **H25, H43, H44, H34**), 7.91 (1 H, s, **H6**), 7.86–7.82 (1 H, m, **H42**), 7.70 (1 H, d, *J* = 8.5 Hz, **H31**), 7.63–7.57 (1 H, m, **H32**), 7.44–7.33 (4 H, m, **H14, H33, H38**), 7.29–7.20 (6 H, m, **H9, H15**), 7.19–7.13 (1 H, m, **H16**), 6.89 (1 H, s, **H36**), 6.85–6.81 (4 H, m, **H10**), 6.10 (1 H, t, *J*= 6.6 Hz, **H1′**), 5.33 (1 H, d, *J* = 4.4 Hz, **OH**), 4.69–4.59 (4 H, m, **H26, H28**), 4.29–4.22 (1 H, m, **H3′**), 4.18 (3 H, s, **H46**), 3.93–3.88 (1 H, m, **H4′**), 3.68 (6 H, s, **H12**), 3.66 (2 H, s, **H23**), 3.35 (2 H, s, **H19**), 3.28 (2 H, d, *J* = 2.1 Hz, **H20**), 3.22 (1H, t, *J* = 2.1 Hz, **H22**), 3.21–3.18 (1 H, m, **H5′a**), 3.07 (1 H, dd, *J* = 10.4, 2.4 Hz, **H5′b**), 2.43 (2 H, app. quin., *J*= 7.4 Hz, **H27**), 2.31–2.22 (1 H, m, **H2′a**), 2.22–2.13 (1 H, m, **H2′b**).

^13^C NMR (126 MHz, DMSO-*d*_6_): δ 162.1 (**C2**), 159.7 (**C29**), 158.5 (**C4**), 149.8 (**C^Ar^**), 149.3 (**C39**), 145.7 (**C37**), 145.1 (**C^Ar^**), 143.9 (**C^Ar^**), 143.3 (**C6**), 140.3 (**C30**), 138.4 (**C45**), 136.1 (**C^Ar^**), 135.7 (**C24**), 133.8 (**C43**), 130.2 (**C9**), 128.7 (**C32**), 128.4 (**C15**), 128.0 (**C14**), 127.7 (**C42**), 127.1 (**C16**), 126.0 (**C41**), 125.0 (**C33**), 124.8 (**C25**), 124.5 (**C40**), 124.4 (**C35**), 123.5 (**C34**), 118.8 (**C44**), 113.7 (**C10**), 113.0 (**C31**), 108.7 (**C38**), 98.9 (**C5**), 88.6 (**C7**), 87.7 (**C36**), 86.3 (**C4′**), 86.2 (**C17**), 85.5(**C1′**), 79.5, 77.3 (**C18**, **C21**), 76.5 (**C22**), 70.9 (**C3′**), 64.1 (**C5′**), 55.5 (**C12**), 47.7 (**C23**), 47.2 (**C26**), 43.9 (**C28**), 43.0 (**C46**), 42.7 (**C19**), 41.7 (**C20**), 40.1 (**C2′**), 27.2 (**C27**).

**LRMS** [ESI^+^, MeOH] *m/z* (%): 1033.4 ([M-I]^+^, 100%).

**HRMS** [ESI^+^, MeOH] calc. for C_60_H_57_O_7_N_8_S, [M-I]^+^, 1033.4065, found 1033.4028.

**5´-DMT Thiazole Orange C8 dU**

*N*-(8-Azidooctyl)-2-[(1,4-dihydro-1-methylquinolin-4-ylidene)methyl] benzothiazolium iodide **3-TO8** (550 mg, 0.96 mmol, 1.0 eq.) and 5′-*O*-(4,4′-dimethoxytrityl)-5-[(*N,N*-dipropyn-2-yl)-3-aminopropyn-1-yl)]-2′-deoxyuridine **4** (763 mg, 1.15 mmol, 1.2 eq.) were dissolved in DMF (10 mL), to which was added tris(benzyltriazolylmethyl)amine (TBTA) (256 mg, 0.48 mmol, 0.5 eq.). A solution of sodium ascorbate (382 mg, 1.93 mmol, 2.0 eq.) in water (1 mL) was added, followed by a solution of copper(II) sulfate (120 mg, 0.48 mmol, 0.5 eq.) in water (1 mL). The reaction mixture was stirred at room temperature for 2 h, the solvent was removed under reduced pressure, and the residue was dissolved in 10% MeOH in DCM (25 mL). The mixture was washed with a 5% Na_2_EDTA solution (25 mL × 3), then with distilled H_2_O (30 mL × 2) and brine (30 mL × 2). The organic layer was dried over anhydrous Na_2_SO_4_ then filtered, and the solvent was concentrated under reduced pressure. Following purification by column chromatography (1–10% MeOH/DCM, 0.5% pyridine) compound **5‑TO8** was obtained as an orange foam (405 mg, 0.28 mmol, 29%).

R_f_: 0.18 (EtOAc/MeOH/conc. aq. NH_3_ 5:1:1)

^1^H NMR (400 MHz, DMSO-*d*_6_): δ 11.69 (1 H, s, **NH**), 8.73 (1 H, d, *J* = 8.6 Hz, **H46**), 8.63–8.60 (1 H, m, **H42**), 8.08–7.97 (3 H, m, **H48, H49, H39**), 7.91 (1 H, s, **H25**), 7.90 (1 H, s, **H6**), 7.81–7.73 (2 H, m, **H47, H36**), 7.62–7.56 (1 H, m, **H37**), 7.41–7.34 (3 H, m, **H43, H14**), 7.28–7.22 (7 H, m, **H38, H9, H15**), 7.19–7.13 (1 H, m, **H16**), 6.93 (1 H, s, **H41**), 6.87–6.81 (4 H, m, **H10**), 6.13–6.07 (1 H, m, **H1′**), 5.34 (1 H, d, *J* = 4.6 Hz, **OH**), 4.61 (2 H, t, *J* = 7.1 Hz, **H33**), 4.30–4.21 (3 H, m, **H3′, H26**), 4.17 (3 H, s, **H51**), 3.92–3.87 (1 H, m, **H4′**), 3.69 (6 H, s, **H12**), 3.61 (2 H, s, **H23**), 3.32 (2 H, s, **H19**), 3.26 (2 H, br. s, **H20**), 3.24–3.18 (2 H, m, **H22**, **H5′a**), 3.09–3.03 (1 H, m, **H5′b**), 2.34–2.12 (2 H, m, **H2′**), 1.83–1.66 (4 H, m, **H32, H27**), 1.56–1.20 (8 H, m, **H28, H29, H30, H31**).

^13^C NMR (101 MHz, DMSO-*d*_6_): δ 162.2 (**C2**), 159.7 (**C34**), 158.5 (**C4**), 149.6, 149.2 (**C44**, **C^Ar^**), 145.6 (**C42**), 145.2 (**C^Ar^**), 143.7 (**C^Ar^**), 144.1 (**C6**), 140.5 (**C35**), 138.4 (**C50**), 136.6 (**C^Ar^**), 135.6 (**C24**), 133.8 (**C48**), 130.1 (**C9**), 128.7 (**C37**), 128.3 (**CH^Ar^**), 128.0 (**CH^Ar^**), 127.7 (**C47**), 127.1 (**C16**), 125.8 (**C46**), 124.9 (**C38**), 124.6 (**C25**), 124.4 (**C40**), 124.4 (**C45**), 123.4 (**C39**), 118.9 (**C49**), 113.7 (**C10**), 113.5 (**C36**), 108.5 (**C43**), 99.5 (**C5**), 88.7 (**C7**), 88.4 (**C41**), 86.3 (**C4′**), 86.3 (**C17**), 85.5 (**C1′**), 79.0, 77.3 (**C21**, **C18**), 76.3 (**C22**), 70.9 (**C3′**), 64.0 (**C5′**), 55.7 (**C12**), 50.1 (**C26**), 47.8 (**C23**), 46.0 (**C33**), 42.6 (**C51**), 42.1, 41.8 (**C19**, **C20**), 40.5 (**C2′**), 29.0, 28.9, 28.7 (**C27**, **C28**, **C29**), 26.7 (**C32**), 26.4 (**C30**), 26.1 (**C31**).

**LRMS** [ESI^+^, MeOH] *m/z* (%): 1103.4 ([M-I]^+^, 100%).

HRMS [ESI^+^, MeOH] calc. for C_65_H_67_O_7_N_8_S, [M-I]^+^, 1103.4848, found 1103.4812.

**Thiazole Orange C3 dU phosphoramidite monomer**

5′-*O*-(4,4′-Dimethoxytrityl)-5-[(*N*-(1-(3-(2-((1,4-dihydro-1-methylquinolin-4-ylidene)methyl)benzothiazolium)propyl)triazol-4-yl-methyl))-*N*-(propyn-2-yl)-3-aminopropyn-1-yl)]-2′-deoxyuridine iodide **5-TO3** (240 mg, 207 µmol, 1.0 eq.) was co-evaporated 3 times with anhydrous pyridine and dried overnight under reduced pressure, then co-evaporated with anhydrous DCM (5 mL × 3) and dried again under reduced pressure. After 1 h the compound **5-TO3** was dissolved in anhydrous DCM (3 mL) under an atmosphere of dry argon with a few drops of anhydrous DMF to improve the solubility. Anhydrous DIPEA (108 µL, 621 µmol, 3.0 eq.) was added, followed by addition of 2-cyanoethyl-*N,N*‑diisopropylchlorophosphoramidite (116 µL, 518 µmol, 2.5 eq.) dropwise. The reaction mixture was stirred at room temperature for 2 h and transferred under argon into a separating funnel containing degassed DCM (15 mL). The mixture was washed with degassed saturated aqueous KCl (20 mL) and the organic layer was separated, dried over anhydrous Na_2_SO_4_ then filtered, and concentrated under reduced pressure. The crude phosphoramidite product was reverse precipitated by dissolving in anhydrous DCM (1 mL) and adding anhydrous hexane (20 mL). After purification by repeated reverse precipitation (×3), and the product **6-TO3** was isolated as a dark orange foam (195 mg, 143 µmol, 70%).

R_f_: 0.29 (10% MeOH/DCM, 0.5% pyridine)

^1^H NMR (500 MHz, CD_2_Cl_2_-*d*_2_): δ 8.70–8.63 (1 H, m, **H41**), 8.51 (1 H, d, *J* = 7.3 Hz, **H37**), 8.02 (1 H, s, **H25**), 7.98 (1 H, s, **H6**), 7.80–7.74 (2 H, m, **H44, H34**), 7.62 (1 H, d, *J*= 7.9 Hz, **H43**), 7.60–7.56 (1 H, m, **H42**), 7.47–7.41 (4 H, m, **H31, H32, H33, H38**), 7.37–7.27 (8 H, m, **H14**, **H15**, **H9**), 7.24–7.18 (1 H, m, **H16**), 6.87–6.82 (4 H, m, **H10**), 6.71 (1 H, s, **H36**), 6.25 (1 H, dd, *J* = 12.7, 6.3 Hz, **H1′**), 4.78–4.70 (2 H, m, **H28**), 4.67–4.52 (3 H, m, **H26, H3′**), 4.22–4.13 (1 H, m, **H4′**), 4.09 (3 H, s, **H46**), 3.86–3.78 (2 H, m, **H47**), 3.76 (6 H, s, **H12**), 3.73–3.64 (2 H, m, **H50**), 3.63–3.52 (3 H, m, **H5′**, **H22**), 3.40–3.31 (4 H, m, **H23, H19**), 3.30–3.25 (2 H, m, **H20**), 2.63 (1 H, t, *J* = 6.2 Hz, **H48a**), 2.61–2.50 (3 H, m, **H2′a**, **H27**), 2.48 (1 H, t, *J* = 6.2 Hz, **H48b**), 2.37–2.27 (1 H, m, **H2′b**), 1.20–1.15 (9 H, m, **H51**), 1.09 (3 H, d, *J* = 6.6 Hz, **H51**).

^31^P NMR (202 MHz, CD_2_Cl_2_-*d*_2_): δ 149.10, 148.92.

**Thiazole Orange C8 dU phosphoramidite monomer**

5′-*O*-(4,4′-Dimethoxytrityl)-5-[(*N*-(1-(8-(2-((1,4-dihydro-1-methylquinolin-4-ylidene) methyl)benzothiazolium)octyl)triazol-4-yl-methyl))-*N*-(propyn-2-yl)-3-aminopropyn-1-yl)]-2′-deoxyuridine iodide **5-TO8** (202 mg, 164 µmol, 1.0 eq.) was co-evaporated with anhydrous pyridine (5 mL × 3) and dried overnight under reduced pressure, co‑evaporated with anhydrous DCM (5 mL × 3) and dried again under reduced pressure. After 1 h the compound **5-TO8** was dissolved in anhydrous DCM (3 mL) under an atmosphere of argon with a few drops of anhydrous DMF to improve the solubility, then anhydrous DIPEA (70 µL, 400 µmol, 2.5 eq.) was added, followed by addition of 2-cyanoethyl-*N,N*‑diisopropylchlorophosphoramidite (73 µL, 328 µmol, 2.0 eq) dropwise. The reaction mixture was stirred at room temperature for 2 h and transferred under argon into a separating funnel containing degassed DCM (15 mL). The mixture was washed with degassed saturated aqueous KCl (20 mL) and the organic layer was separated, dried over anhydrous Na_2_SO_4_ then filtered, and concentrated under reduced pressure. The crude phosphoramidite product was purified by reverse precipitation by dissolving in anhydrous DCM (1 mL) and adding anhydrous hexane (20 mL). After repeated reverse precipitation (×3), the product **6-TO8** was isolated as a dark orange foam (155 mg, 108 µmol, 66%).

R_f_: 0.52 (10% MeOH/DCM, 0.5% pyridine)

^1^H NMR (400 MHz, CD_2_Cl_2_-*d*_2_): δ 8.81 (1 H, d, *J* = 6.9 Hz, **H42**), 8.42 (1 H, d, *J*= 8.3 Hz, **H46**), 7.97 (1 H, s, **H25**), 7.93 (1 H, s, **H6**), 7.93–7.88 (1 H, m, **H48**), 7.86–7.76 (2 H, m, **H49, H39**), 7.74–7.68 (1 H, m, **H47**), 7.53 (1 H, d, *J* = 6.3 Hz, **H36**), 7.47–7.38 (4 H, m, **H37, H43, H14**), 7.35–7.25 (7 H, m, **H38, H9, H15**), 7.22–7.16 (1 H, m, **H16**), 6.86–6.80 (4 H, m, **H10**), 6.72 (1 H, s, **H41**), 6.30–6.18 (1 H, m, **H1′**), 4.63–4.56 (1 H, m, **H3′**), 4.40 (2 H, t, *J* = 7.2 Hz, **H33**), 4.29–4.25 (2 H, m, **H26**), 4.24 (3 H, s, **H51**), 4.21–4.17 (1 H, m, **H4′**), 3.75 (6 H, s, **H12**), 3.73–3.66 (4 H, m, **H52**, **H55**), 3.65–3.51 (3 H, m, **H22**, **H5′**), 3.36–3.27 (4 H, m, **H23, H19**), 3.25–3.21 (2 H, m, **H20**), 2.64–2.49 (2 H, m, containing t, *J* = 6.2 Hz, **H53a**, **H2′a**), 2.46 (1 H, t, *J* = 6.2 Hz, **H53b**), 2.33–2.26 (1 H, m, **H2′b**), 1.92 (2 H, app. quin., *J*= 7.6 Hz, **H32**), 1.83 (2 H, app. quin., *J*= 7.0 Hz, **H27**), 1.67–1.36 (8H, m, **H28, H29, H30, H31**), 1.18–1.11 (9 H, m, **H56**), 1.08 (3 H, d, *J*= 6.9 Hz, **H56**).

^31^P NMR (162 MHz, CD_2_Cl_2_-*d*_2_): δ 148.73, 148.64.

**1-Methyl-4-chloropyridinium iodide**

4-Chloropyridine hydrochloride (10.0 g, 66.7 mmol, 1 eq.) was dissolved in H_2_O (10 mL). Excess saturated NaHCO_3_ solution was added until pH 7 was reached. DCM (100 mL) was added to extract 4-chloropyridine. The orangic layer was collected, then concentrated under vacuum (care, compound is volatile). Iodomethane (10.0 mL, 240 mmol, 3.6 eq.) was added to the reaction in a 100 mL flask, and the mixture was left standing in the dark at 4 °C for 3 days. The precipitate was collected and washed with cold Et_2_O (30 mL × 3), then dried under reduced pressure to give product **S5** as a brown solid([7](#_ENREF_7)) (10.3 g, 40.4 mmol, 61%).

R_f_: 0.25 (10% MeOH/DCM)

^1^H NMR (400 MHz, DMSO-*d*_6_): δ 9.00 (2 H, d, *J* = 6.9 Hz, **H2**) 8.35 (2 H, d, *J*= 6.9 Hz, **H3**) 4.29 (3 H, s, **H5**).

^13^C NMR (101 MHz, DMSO-*d*_6_): δ 152.1 (**C4**), 147.2 (**C2**), 128.4 (**C3**), 47.5 (**C5**).

**LRMS** [ESI^+^, MeOH] *m/z* (%): 128.1 ([M-I]^+^, 100%).

***N*-(3-Iodopropyl)-2-[(1,4-dihydro-1-methylpyridin-4-ylidene)methyl]benzothiazolium iodide**

*N*-(3-Iodopropyl)-2-methylbenzothiazolium iodide **2-3** (3.18 g, 7.15 mmol, 1.0 eq.) and 1-methyl-4‑chloropyridium iodide **S5** (3.64 g, 14.3 mmol, 2.0 eq.) were dissolved in DCM/MeCN (1:1, 50 mL) under an atmosphere of dry argon. Anhydrous Et_3_N (2.50 mL, 17.9 mmol, 2.5 eq.) was added, producing an immediate deep red color. The reaction mixture was stirred for 10 min at room temperature. The solvent was removed under reduced pressure and the residue was washed with cold Et_2_O (50 mL × 3) and dried under reduced pressure. The multi-component residue was purified by column chromatography (1–10% MeOH/DCM) to give the compound **S6** as a yellow solid (0.40 g, 0.87 mmol, 10%).

R_f_: 0.30 (EtOAc/MeOH/conc. aq. NH_3_ 5:1:1)

^1^H NMR (400 MHz, DMSO-*d*_6_): δ 8.33 (2 H, d, *J* = 7.1 Hz, **H2′**), 7.92 (1 H, d, *J*= 7.5 Hz, **H7**), 7.61 (1 H, d, *J*= 8.4 Hz, **H4**), 7.53 (1 H, t, *J* = 7.5 Hz, **H5**), 7.42 (2 H, d, *J*= 7.1 Hz, **H3′**), 7.31 (1 H, t, *J* = 7.5 Hz, **H6**), 6.31 (1 H, s, **H2a**), 4.29 (2 H, t, *J*= 7.2 Hz, **H8**), 4.00 (3 H, s, **H5′**), 3.39 (2 H, t, *J* = 7.2 Hz, **H10**), 2.25 (2 H, app. quin., *J*= 7.2 Hz, **H9**).

^13^C NMR (126 MHz, DMSO-*d_6_*): δ 153.3 (**C2**), 150.8 (**C4′**), 142.9 (**C2′**), 140.9 (**C3a**), 127.7 (**C5**), 124.2 (**C6**), 123.1 (**C7a**), 122.6 (**C7**), 117.4 (**C3′**), 111.5 (**C4**), 97.2 (**C2a**), 45.3 (**C5′**), 44.7 (**C8**), 25.2 (**C10**), 20.7 (**C9**).

**LRMS** [ESI^+^, MeOH] *m/z* (%): 409.0 ([M-I]^+^, 100%).

**HRMS** [ESI^+^, MeOH] calc. for C_17_H_18_N_2_IS, [M-I]^+^, 409.0230, found 409.0230.

***N*-(3-Azidopropyl)-2-[(1,4-dihydro-1-methylpyridin-4-ylidene)methyl] benzothiazolium iodide**

*N*-(3-Iodopropyl)-2-[(1,4-dihydro-1-methylpyridin-4-ylidene)methyl]benzothiazolium iodide **S6** (0.20 g, 0.37 mmol, 1 eq.) was dissolved in DMF (10 mL). Lithium azide solution (20% in water, 0.22 mL, 0.93 mmol, 2.5 eq.) was added dropwise and the reaction mixture was stirred at room temperature for 10 min. The solvent was removed under reduced pressure and the residue was purified by column chromatography (1–10% MeOH/DCM) to give **7-BO3** as a yellow solid (125 mg, 0.28 mmol, 76%).

R_f_: 0.29 (EtOAc/MeOH/conc. aq. NH_3_ 5:1:1)

^1^H NMR (400 MHz, DMSO-*d*_6_): δ 8.32 (2 H, d, *J* = 7.2 Hz, **H2′**), 7.92 (1 H, dd, *J*= 7.8, 1.0 Hz, **H7**), 7.59 (1 H, d, *J*= 8.1 Hz, **H4**), 7.57–7.51 (1 H, m, **H5**), 7.44 (2 H, d, *J* = 7.2 Hz, **H3′**), 7.35–7.29 (1 H, m, **H6**), 6.30 (1 H, s, **H2a**), 4.32 (2 H, t, *J*= 7.2 Hz, **H8**), 4.00 (3 H, s, **H5′**), 3.54 (2 H, t, *J* = 6.6 Hz, **H10**), 1.99 (2 H, app. quin., *J*= 7.1 Hz, **H9**).

^13^C NMR (101 MHz, DMSO-*d_6_*): δ 156.7 (**C2**), 150.7 (**C4′**), 142.8 (**C2′**), 140.4 (**C3a**), 128.2 (**C5**), 124.1 (**C6**), 123.8 (**C7a**), 123.1 (**C7**), 119.0 (**C3′**), 112.2 (**C4**), 89.6 (**C2a**), 48.2 (**C10**), 45.5 (**C5′**), 43.1 (**C8**), 26.2 (**C9**).

**LRMS** [ESI^+^, MeOH] m/z (%): 324.1 ([M-I]^+^, 100%).

**HRMS** [ESI^+^, MeOH] calc. for C_17_H_18_N_5_S, [M-I]^+^, 324.1277, found 324.1275.

**5´-DMT benzothiazole orange C3 dU**

*N*-(3-Azidopropyl)-2-[(1,4-dihydro-1-methylpyridin-4-ylidene)methyl]benzothiazolium iodide **7-BO3** (340 mg, 0.75 mmol, 1.0 eq.) and 5′-*O*-(4,4′-dimethoxytrityl)-5-[(*N,N*-dipropyn-2-yl)-3-aminopropyn-1-yl)]-2′-deoxyuridine **4** (646 mg, 0.98 mmol, 1.3 eq.) were dissolved in DMF (8 mL), to which solution was added tris(benzyltriazolylmethyl)amine (TBTA) (200 mg, 0.37 mmol, 0.5 eq.) and a solution of sodium ascorbate (299 mg, 1.5 mmol, 2.0 eq.) in water (1 mL), followed by a solution of copper(II) sulfate (94.1 mg, 0.37 mmol, 0.5 eq.) in water (1 mL). The reaction mixture was stirred at room temperature for 2 h. The solvent was removed under reduced pressure, and the residue was dissolved in 10% MeOH in DCM (25 mL). The mixture was washed with a 5% Na_2_EDTA solution (25 mL × 3), then with distilled H_2_O (30 mL × 2) and brine (30 mL × 2). The organic layer was dried over anhydrous Na_2_SO_4_ then filtered, and concentrated under reduced pressure. Following purification of the complex mixture by column chromatography (1–10% MeOH/DCM, 0.5% pyridine), **8-BO3** was afforded as a yellow foam (250 mg, 0.25 mmol, 26%).

R_f_: 0.25 (10% MeOH/DCM, 0.5% pyridine)

^1^H NMR (400 MHz, DMSO-*d*_6_): δ 11.69 (1 H, s, **NH**), 8.32 (2 H, d, *J* = 7.0 Hz, **H37**), 7.95 (1 H, s, **H25**), 7.92 (1 H, s, **H6**), 7.90 (1 H, d, *J* = 7.8 Hz, **H34**), 7.51–7.47 (2 H, m, **H31, H32**), 7.42–7.33 (4 H, m, **H38, H14**), 7.32–7.22 (7 H, m, **H33, H9, H15**), 7.18 (1 H, t, *J* = 7.2 Hz, **H16**), 6.87–6.82 (4 H, m, **H10**), 6.17 (1 H, s, **H36**), 6.10 (1 H, t, *J*= 6.6 Hz, **H1′**), 5.33 (1 H, d, *J* = 4.3 Hz, **OH**), 4.54 (2 H, t, *J* = 6.8 Hz, **H26**), 4.33–4.22 (3 H, m, **H28, H3′**), 3.98 (3 H, s, **H40**), 3.93–3.88 (1 H, m, **H4′**), 3.69 (6 H, s, **H12**), 3.64 (2 H, s, **H23**), 3.35 (2 H, s, **H19**), 3.28 (2 H, br. s, **H20**), 3.25–3.18 (2 H, m, **H5′a, H22**), 3.12–3.04 (1 H, m, **H5′b**), 2.36–2.13 (4 H, m, **H27, H2′**).

^13^C NMR (126 MHz, DMSO-*d*_6_): δ 162.0 (**C2**), 158.6 (**C4**), 156.6 (**C29**), 150.8 (**C39**), 149.8 (**C^Ar^**), 145.2 (**C^Ar^**), 143.9 (**C^Ar^**), 143.3 (**C6**), 142.7 (**C37**), 140.2 (**C30**), 136.0 (**C^Ar^**), 135.7 (**C24**), 130.1 (**C9**), 128.4, 128.3 (**C32**, **C15**), 128.0 (**C14**), 127.1 (**C16**), 124.6 (**C25**), 124.1 (C**33**), 123.8 (**C35**), 123.1 (**C34**), 119.0 (**C38**), 113.7 (**C10**), 112.1 (**C31**), 98.9 (**C5**), 89.5 (**C36**), 88.6 (**C7**), 86.4, 86.3 (**C4′**, **C17**), 85.5 (**C1′**), 79.4, 77.3 (**C18**, **C22**), 76.5 (**C21**), 70.9 (**C3′**), 64.1 (**C5′**), 55.5 (**C12**), 47.6 (**C23**), 47.2 (**C26**), 45.5 (**C40**), 43.0 (**C28**), 42.6 (**C19**), 41.7 (**C20**), 40.2 (**C2′**), 27.1 (**C27**).

**LRMS** [ESI^+^, MeOH] *m/z* (%): 681.3 ([M-I-DMT+H]^+^, 100%); 303.1 ([DMT]^+^, 100%).

**HRMS** [ESI^+^, MeOH] calc. for C_56_H_55_O_7_N_8_S, [M-I]^+^, 983.3909, found 983.3866.

**5´-DMT benzothiazole orange C3 dU phosphoramidite monomer**

5′-*O*-(4,4′-Dimethoxytrityl)-5-[(*N*-(1-(3-(2-((1,4-dihydro-1-methylpyridin-4-ylidene)methyl)benzothiazolium)propyl)triazol-4-yl-methyl))-*N*-(propyn-2-yl)-3-aminopropyn-1-yl)]-2′-deoxyuridine iodide **8-BO3** (250 mg, 225 µmol, 1.0 eq.) was co-evaporated with anhydrous pyridine (5 mL × 3) and dried overnight under reduced pressure, then co‑evaporated with anhydrous DCM (5 mL × 3) and dried again under reduced pressure. After 1 h the compound **8-BO3** was dissolved in anhydrous DCM (3 mL) under an atmosphere of dry argon with a few drops of anhydrous DMF to improve the solubility, then anhydrous DIPEA (98 µL, 562 µmol, 2.5 eq.) was added, followed by addition of 2-cyanoethyl-*N,N*‑diisopropylchlorophosphoramidite (100 µL, 450 µmol, 2.0 eq.) dropwise. The reaction mixture was stirred at room temperature for 2 h and transferred under argon into a separating funnel containing degassed DCM (15 mL). The mixture was washed with degassed saturated aqueous KCl (20 mL) and the organic layer was separated, dried over anhydrous Na_2_SO_4_, filtered, and concentrated under reduced pressure. The crude phosphoramidite product was purified by reverse precipitation by dissolving in anhydrous DCM (1 mL) and adding anhydrous hexane (20 mL). After repeated reverse precipitation (×3), the **9-BO3** was isolated as a yellow foam (190 mg, 145 µmol, 64%).

R_f_: 0.42 (10% MeOH/DCM, 0.5% pyridine)

^1^H NMR (400 MHz, DMSO-*d*_6_): δ 11.67 (1 H, br. s., **NH**), 8.32 (2 H, d, *J* = 6.4 Hz, **H37**), 7.96 (1 H, s, **H25**), 7.93 (1 H, s, **H6**), 7.89 (1 H, d, *J* = 7.7 Hz, **H34**), 7.53–7.45 (2 H, m, **H31, H32**), 7.41–7.33 (4 H, m, **H38**, **H14**), 7.31–7.21 (7 H, m, **H33, H9, H15**), 7.20–7.14 (1 H, m, **H16**), 6.88–6.80 (4 H, m, **H10**), 6.19 (1 H, s, **H36**), 6.14–6.05 (1 H, m, **H1′**), 4.55 (2 H, t, *J* = 6.6 Hz, **H26**), 4.50–4.42 (1 H, m, **H3′**), 4.30 (2 H, t, *J* = 7.3 Hz, **H28**), 4.10–4.01 (1 H, m, **H4′**), 3.99 (3 H, s, **H40**), 3.70 (6 H, s, **H12**), 3.67–3.64 (2 H, m, **H44**), 3.63–3.56 (2 H, m, **H41**), 3.57–3.43 (2 H, m, **H5′**), 3.41–3.09 (7 H, m, **H19**, **H20**, **H22**, **H23**), 2.98–2.84 (1 H, m, **H2′a**), 2.76 (1 H, t, *J* = 5.8 Hz, **H42a**), 2.64 (1 H, t, *J* = 5.8 Hz, **H42b**), 2.46–2.39 (1 H, m, **H2′b**), 2.37–2.24 (2 H, m, **H27**), 1.17–1.05 (9 H, m, **H45**), 0.98 (3 H, d, *J*= 6.6 Hz, **H45**).

^31^P NMR (162 MHz, DMSO-*d*_6_): δ 147.58, 147.19.

## Oligonucleotide Synthesis and Purification

### General

Standard DNA phosphoramidites, solid supports (controlled pore glass, CPG or polystyrene) and additional reagents were purchased from Link Technologies, Glen Research and Applied Biosystems. NAP Sephadex gel‑filtration columns were purchased from GE Healthcare and used according to the manufacturer’s instructions. All oligonucleotides were synthesized on an Applied Biosystems 394 automated DNA/RNA synthesizer using a standard 1.0 μmol phosphoramidite cycle of acid-catalysed detritylation, coupling, capping, and iodine oxidation. Stepwise coupling efficiencies and overall yields were determined by automated trityl cation conductivity monitoring and in all cases were >98.0%. All β‑cyanoethyl phosphoramidite monomers except for **6-TO3**, **6‑TO8** and **9-BO3** monomers (which were dissolved in anhydrous DCM) were dissolved in anhydrous acetonitrile to a concentration of 0.1 M immediately prior to use. Standard DNA phoshoramidites were used for the majority of oligonucleotide sequences. For oligonucleotides containing **6-TO3**, **6-TO8** and **9-BO3**, fast deprotecting monomers were used (UltraMILD monomers, Pac-dA, Ac-dC and iPr‑Pac‑dG, Link Technologies). The coupling time for A, G, C, and T monomers was 60 s, and the coupling time for the modified phosphoramidite monomers (**6-TO3**, **6-TO8** and **9-BO3**) was extended to 600 s. Compounds **S4** and **S7** (octadiynyl dU phosphoramidite, Scheme S1) were used for the introduction of the alkyne functional groups into oligonucleotides and coupled for 600 s.

Cleavage of the oligonucleotides from the solid support and subsequent deprotection was achieved by exposure to concentrated aqueous ammonia solution for 1 h at room temperature, followed by heating in a sealed tube for 5 h at 55 °C. In the case of oligonucleotides containing **6-TO3**, **6-TO8** and **9-BO3** deprotection was carried out for 4 h at room temperature or 1 h at 55 °C.

The oligonucleotides were purified by reversed-phase HPLC on a Gilson system using a Luna 10 μm C8 100 Å pore Phenomenex column (10 × 250 mm) with a gradient of acetonitrile in ammonium acetate or triethylammonium bicarbonate (TEAB) over 20 min at a flow rate of 4 mL min^-1^. TEAB buffers (buffer A: 0.1 M triethylammonium bicarbonate, pH 7.5; buffer B: 0.1 M triethylammonium bicarbonate, pH 7.5, with 50% acetonitrile) were used (0% to 80% buffer B over 20 min). Elution was monitored by UV absorption at 295 nm. After HPLC purification, all oligonucleotides were characterised by electrospray mass spectrometry using a Bruker micrOTOF II focus ESI-TOF MS instrument in ESI^-^ mode. Data were processed using MaxEnt.

Scheme S1. Preparation of Non-Intercalative Control Probes

Conditions: (i) solid phase oligonucleotide synthesis; (ii) DMSO, H_2_O, tris(benzyltriazolylmethyl)amine (TBTA_, Na ascorbate, CuSO_4_.5H_2_O, 55^o^ C, 2 h.

### General Method for Solid phase Labelling of Oligonucleotides with Fluorescent Dyes by the CuAAC Reaction

A solution of Cu^I^ click catalyst was prepared from tris(3-hydroxypropyltriazolylmethyl) amine ligand (THPTA, 2.1 μmol for one addition, 35 eq.; 4.2 μmol for two additions, 70 eq.; both in H_2_O, 5.2 μL), sodium ascorbate (3.0 μmol for one addition, 50 eq.; 6.0 μmol for two additions, 100 eq.; both in H_2_O, 3.2 μL) and CuSO_4_∙5H_2_O (0.3 μmol for one addition, 5 eq., 0.6 μmol for two additions, 10 eq., both in H_2_O, 1.6 μL). The Cu^I^ solution was mixed with the fluorescent dye azide (0.6 μmol, 10 eq. for one addition; 0.9 μmol, 15 eq. for two additions) in DMSO (20 μL) . The mixture was added to the TO- or BO-modified oligonucleotide on the solid support (60 nmol). All CuAAC reactions were carried out in PCR tubes (250 μL, sealed with parafilm) and heated in a heating block at 55 °C for 4 h. After the click reaction, the solid support was washed with H_2_O and acetonitrile (1 mL × 3), then dried by the passage of a stream of argon gas. The resultant ligated oligonucleotide was cleaved from the solid support and deprotected by treating with concentrated aqueous ammonia solution for 4 h at room temperature in a sealed tube. All ligated oligonucleotide products were analysed by reversed-phase HPLC and characterised by mass spectrometry.

**Table S1.** Modified Nucleoside Structures (R = DNA or RNA).

| **X** | **Dye code** | **Structure** |
| --- | --- | --- |
| **X_1_** | **No dye** |  |
| **X_2_** | **Dye = TO3** |  |
| **X_3_** | **Dye = TO8** |  |
| **X_4_** | **Dye = BO3** |  |
| **X_5_** | **Dye = ROX** |  |
| **X_6_** | **Dye = ROX** |  |
| **X_7_** | **Dye = FAM** |  |
| **X_8_** | **Dye = FAM-C6** |  |
| **X_9_** | **Dye = HEX** |  |
| **X_10_** | **Dye = ATTO647N** |  |
| **X_11_** | **Dye1 = TO3, Dye2 = TO3** |  |
| **X_12_** | **Dye1 = TO3, Dye2 = TO8** |  |
| **X_13_** | **Dye1 = TO8, Dye2 = TO8** |  |
| **X_14_** | **Dye1 = TO3, Dye2 = ROX** |  |
| **X_15_** | **Dye1 = TO8, Dye2 = ROX** |  |
| **X_16_** | **Dye1 = TO3, Dye2 = FAM** |  |
| **X_17_** | **Dye1 = TO3, Dye2 = FAM-C6** |  |
| **X_18_** | **Dye1 = TO3, Dye2 = HEX** |  |
| **X_19_** | **Dye1 = TO3, Dye2 = ATTO647N** |  |
| **X_20_** | **Dye1 = BO3, Dye2 = ROX** |  |
| **X_21_** | **Dye1 = BO3, Dye2 = FAM** |  |
| **X_22_** | **Dye1 = BO3, Dye2 = HEX** |  |
| **X_23_** | **Dye1 = BO3, Dye2 = ATTO647N** |  |

All dyes structures are in Figure 2 (main paper) and FAM-C6 is the same structure as FAM but with a C6 linker instead of C3.

### UV Melting Analysis

All UV melting measurements were made on a Varian Cary 4000 UV-VIS spectrophotometer with Cary temperature controller. Cary Win UV Thermal software was used with an absorption wavelength of 260 nm. Samples were analysed in 1 mL cuvettes (Hellma synthetic quartz ‘precision cell QG’; 1 cm pathlength) and were made to 1.25 μM oligonucleotide concentration (total volume = 800 µL) in phosphate buffer (NaH_2_PO_4_, 10 mM) with a total of 200 mM NaCl at pH 7.4. The samples were initially denatured by heating to 85 °C at 10 °C min^-1^ then cooled to 16 °C at 1 °C min^-1^ and maintained at 16 °C for 2 min before heating to 85 °C at 1 °C min^-1^. UV absorption was recorded every 0.5 °C. The melting temperature (T_m_) values were derived from the derivatives of melting curves and calculated at 260 nm using Cary Win UV Thermal application software. Three successive melting curves were measured and averaged.

Table S2. Single and Double Dye-Modified Hybridization Probes and their Duplex Melting Temperatures.

| Probe/Target sequences | Entry | Code | T_m_ (°C) | ΔT_m_ (°C) |
| --- | --- | --- | --- | --- |
|   5ʹ–TCATCCTATTCTC–3ʹ  Target: 3ʹ–AGTAGGATAAGAG–5ʹ | 1 | S-control  (S-complement) | 43.8 | – |
|   5ʹ–TCATCCTAXTCTC–3ʹ | 2 | S-alkyne: A = alkyne; B = alkyne | 44.1 | +0.3 |
|  | 3 | S-TO3: A = TO3; B = alkyne | 55.4 | +11.6 |
|  | 4 | S-TO8: A = TO8; B = alkyne | 53.0 | +9.2 |
|  | 5 | S-TO3/3: A = TO3; B = TO3 | 53.6 | +9.8 |
|  | 6 | S-TO3/8: A = TO3; B = TO8 | 54.4 | +10.6 |
|  | 7 | S-TO8/8: A = TO8; B = TO8 | 54.3 | +10.5 |
|   5ʹ–CGCTTCTGTATCTATATTCATCP–3ʹ  Target: 3ʹ–TACTGCGAAGACATAGATATAAGTAGTATC–5ʹ | 8 | L-control  (L-complement) | 60.7 | – |
|   5ʹ–CGCTTCXGTATCTAXATTCATCP–3ʹ | 9 | L1-(alkyne)_2_: A = alkyne; B = alkyne | 61.1 | +0.4 |
|  | 10 | L1-(TO3)_2_: A = TO3; B = alkyne | 69.4 | +8.7 |
|  | 11 | L1-(BO3)_2_: A = BO3; B = alkyne | 64.6 | +3.9 |
|  | 12 | L1-(TO3/3)_2_: A = TO3; B = TO3 | 65.4 | +4.7 |
|   5ʹ–CGCTTCXGTATCTATATTCATCP–3ʹ | 13 | L3-alkyne: A = alkyne; B = alkyne | 61.1 | +0.4 |
|  | 14 | L3-TO3: A = TO3; B = alkyne | 66.7 | +6.0 |
|  | 15 | L3-TO8: A = TO8; B = alkyne | 65.2 | +4.5 |
|  | 16 | L3-TO3/3: A = TO3; B = TO3 | 65.5 | +4.8 |
|  | 17 | L3-TO3/8: A = TO3; B = TO8 | 66.2 | +5.5 |
|  | 18 | L3-TO8/8: A = TO8; B = TO8 | 62.4 | +1.7 |
|   5ʹ–CGCTTCTGTATCTAXATTCATCP–3ʹ | 19 | L4-alkyne: A = alkyne; B = alkyne | 60.6 | -0.1 |
|  | 20 | L4-TO3: A = TO3; B = alkyne | 66.0 | +5.3 |
|  | 21 | L4-TO8: A = TO8; B = alkyne | 64.7 | +4.0 |
|  | 22 | L4-TO3/3: A = TO3; B = TO3 | 63.8 | +3.1 |
|  | 23 | L4-TO3/8: A = TO3; B = TO8 | 64.8 | +4.1 |
|  | 24 | L4-TO8/8: A = TO8; B = TO8 | 63.7 | +3.0 |

S = short (13-mer), L = long (22-mer) probe. Red X denotes the position of substitution of a modified thymine base. T_m_ values are the average of 3 separate UV melting and annealing curves monitored at 260 nm in phosphate buffer; NaH_2_PO_4_, 10 mM, 200 mM NaCl at pH 7.4. Melting temperatures are accurate to ±0.2 °C; 3′-P = 3′-propanol which was introduced as a PCR blocker.

**Table S3.** UV Melting Studies of Matched and Mismatched Duplexes.

| Probe strand | Fully  matched  T_m_ (°C) | Mismatch type (Probe:Target) | Mismatched T_m_ (°C) | ΔT_m_ (°C) |
| --- | --- | --- | --- | --- |
| S-TO3 | 55.4 | T:C | 41.9 | –13.5 |
|  |  | T:T | 43.7 | –11.7 |
|  |  | T:G | 46.3 | –9.1 |
| L1-(TO3)_2_ | 69.4 | T:G | 65.9 | –3.5 |
| L3-TO3 | 66.7 | T:G | 63.2 | –3.4 |
| L4-TO3 | 66.0 | T:G | 62.3 | –3.7 |
| L1-(BO3)_2_ | 64.6 | T:G | 61.2 | –3.4 |

T_m_ values are the average of 3 separate UV melting and annealing curves monitored at 260 nm in phosphate buffer; NaH_2_PO_4_, 10 mM, 200 mM NaCl at pH 7.4. ΔT_m_ compares mismatched T_m_ to that of the fully complementary duplex. Melting temperatures are accurate to ±0.2 °C. Sequences are in Table S4.

**UV Melting Analysis with Free Intercalative Dyes**


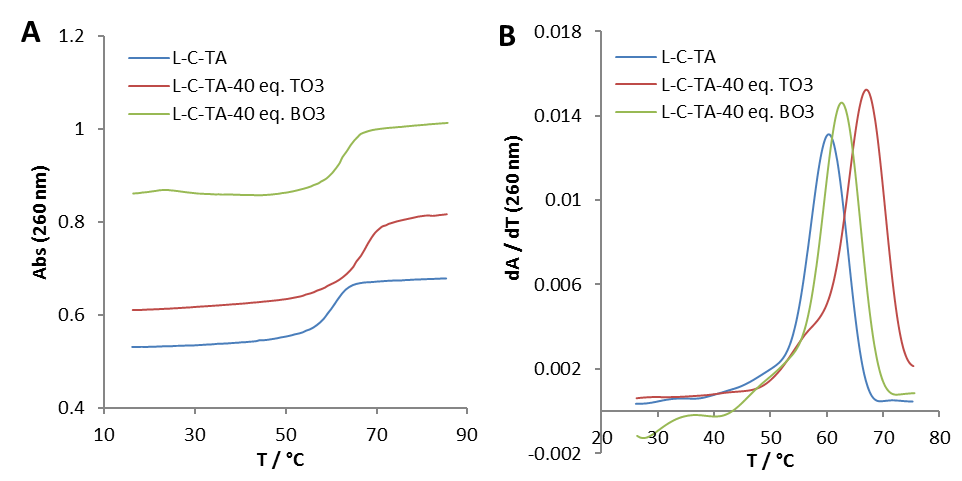


**Figure S1.** Representative UV melting curves (A) their derivatives (B) for oligonucleotides mixed with unattached (free) **3-TO3** and **7-BO3** intercalator dyes. Blue: unmodified T:A matched duplex (L-C-TA-ds) as a control (T_m_ = 60.7 °C); red: duplex after addition of 40 eq. of **3-TO3** (T_m_ = 66.7 °C); green: duplex after addition of 40 eq. of **7‑BO3** (T_m_ = 62.1 °C). L-C-TA = L-control (Table S2, entry 8).

**Table S4.** Oligonucleotide Sequences.

| Code | Sequences | Mass found (calc.) |
| --- | --- | --- |
| S-control | TCATCCTATTCTC | 3836 (3836) |
| S-alkyne | TCATCCTAX_1_TCTC | 3951 (3951) |
| S-TO3 | TCATCCTAX_2_TCTC | 4323 (4325) |
| S-TO8 | TCATCCTAX_3_TCTC | 4394 (4395) |
| S-TO3/3 | TCATCCTAX_11_TCTC | 4697 (4699) |
| S-TO3/8 | TCATCCTAX_12_TCTC | 4767 (4769) |
| S-TO8/8 | TCATCCTAX_13_TCTC | 4837 (4839) |
| S-A Target | GAGAATAGGATGA | 4072 (4072) |
| S-C Target | GAGACTAGGATGA | 4048 (4048) |
| S-T Target | GAGATTAGGATGA | 4063 (4063) |
| S-G Target | GAGAGTAGGATGA | 4087 (4088) |
| S-ROX | TCATCCTAX_5_TCTC | 4567 (4568) |
| S1-TO3/ROX | TCATCCTAX_14_TCTC | 4940 (4942) |
| S-TO8/ROX | TCATCCTAX_15_TCTC | 5011 (5012) |
| S-A Target RNA | GAGAAUAGGAUGA (RNA) | 4251 (4251) |
| S-C Target RNA | GAGACUAGGAUGA (RNA) | 4227 (4227) |
| S-U Target RNA | GAGAUUAGGAUGA (RNA) | 4228 (4228) |
| S-G Target RNA | GAGAGUAGGAUGA (RNA) | 4267 (4267) |
| S2-TO3/ROX | UCAUCCUAX_14_UCUC (RNA) | 5063 (5063) |
| S3-TO3/ROX | UCAUCCUAX_14_UCUC (2ʹ-OMe RNA) | 5231 (5231) |
| L-control | CGCTTCTGTATCTATATTCATCP | 6664 (6664) |
| L1-(alkyne)_2_ | CGCTTCX_1_GTATCTAX_1_ATTCATCP | 6994 (6994) |
| L1-(TO3)_2_ | CGCTTCX_2_GTATCTAX_2_ATTCATCP | 7741 (7742) |
| L1-(BO3)_2_ | CGCTTCX_4_GTATCTAX_4_ATTCATCP | 7641 (7642) |
| L1-(TO3/3)_2_ | CGCTTCX_11_GTATCTAX_11_ATTCATCP | 8488 (8490) |
| L1-(ROX)_2_ | CGCTTCX_6_GTATCTAX_6_ATTCATCP | 8178 (8177) |
| L1-(TO3/ROX)_2_ | CGCTTCX_14_GTATCTAX_14_ATTCATCP | 8975 (8975) |
| L1-(BO3/ROX)_2_ | CGCTTCX_20_GTATCTAX_20_ATTCATCP | 8876 (8875) |
| L1-(FAM)_2_ | CGCTTCX_7_GTATCTAX_7_ATTCATCP | 7861 (7861) |
| L1-(TO3/FAM)_2_ | CGCTTCX_16_GTATCTAX_16_ATTCATCP | 8659 (8659) |
| L1-(BO3/FAM)_2_ | CGCTTCX_21_GTATCTAX_21_ATTCATCP | 8556 (8559) |
| L2-(BO3)_2_ | CGCTTCX_4_GTATCCAX_4_ATTCATCP | 7626 (7626) |
| L2-(FAM-C6)_2_ | CGCTTCX_8_GTATCCAX_8_ATTCATCP | 7930 (7930) |
| L2-(TO3/FAM-C6)_2_ | CGCTTCX_17_GTATCCAX_17_ATTCATCP | 8728 (8728) |
| L2-(HEX)_2_ | CGCTTCX_9_GTATCCAX_9_ATTCATCP | 8260 (8259) |
| L2-(TO3/HEX)_2_ | CGCTTCX_18_GTATCCAX_18_ATTCATCP | 9056 (9057) |
| L2-(BO3/HEX)_2_ | CGCTTCX_22_GTATCCAX_22_ATTCATCP | 8956 (8957) |
| L2-(ATTO647N)_2_ | CGCTTCX_10_GTATCCAX_10_ATTCATCP | 8622 (8621) |
| L2-(TO3/ATTO647N)_2_ | CGCTTCX_19_GTATCCAX_19_ATTCATCP | 9419 (9419) |
| L2-(BO3/ATTO647N)_2_ | CGCTTCX_23_GTATCCAX_23_ATTCATCP | 9318 (9319) |
| L3-TO3 | CGCTTCX_2_GTATCTATATTCATCP | 7251 (7252) |
| L3-TO8 | CGCTTCX_3_GTATCTATATTCATCP | 7322 (7322) |
| L3-ROX | CGCTTCX_5_GTATCTATATTCATCP | 7496 (7495) |
| L3-TO3/ROX | CGCTTCX_14_GTATCTATATTCATCP | 7870 (7869) |
| L3-TO8/ROX | CGCTTCX_15_GTATCTATATTCATCP | 7940 (7939) |
| L3-TO3/3 | CGCTTCX_11_GTATCTATATTCATCP | 7625 (7627) |
| L3-TO3/8 | CGCTTCX_12_GTATCTATATTCATCP | 7696 (7697) |
| L3-TO8/8 | CGCTTCX_13_GTATCTATATTCATCP | 7765 (7766) |
| L4-alkyne | CGCTTCTGTATCTAX_1_ATTCATCP | 6879 (6878) |
| L4-TO3 | CGCTTCTGTATCTAX_2_ATTCATCP | 7251 (7252) |
| L4-TO8 | CGCTTCTGTATCTAX_3_ATTCATCP | 7322 (7322) |
| L4-TO3/3 | CGCTTCTGTATCTAX_11_ATTCATCP | 7625 (7627) |
| L4-TO3/8 | CGCTTCTGTATCTAX_12_ATTCATCP | 7696 (7697) |
| L4-TO8/8 | CGCTTCTGTATCTAX_13_ATTCATCP | 7765 (7766) |
| L4-ROX | CGCTTCTGTATCTAX_5_ATTCATCP | 7497 (7495) |
| L4-TO3/ROX | CGCTTCTGTATCTAX_14_ATTCATCP | 7870 (7869) |
| L4-TO8/ROX | CGCTTCTGTATCTAX_15_ATTCATCP | 7939 (7939) |
| WT Target | CTATGATGAATATAGATACAGAAGCGTCAT | 9262 (9262) |
| MT Target | CTATGATGAATATGGATACAGAAGCGTCAT | 9278 (9278) |
| P1 (PCR primer) | CCTGGCACCATTAAAGAAAATATCATC | 8220 (8220) |
| P2 (PCR primer) | ACATAGTTTCTTACCTCTTCTAGTTGG | 8206 (8206) |
| WT (template for PCR) | CCTGGCACCATTAAAGAAAATATCATCTTTGGTGTTTCCTATGATGAATATAGATACAGAAGCGTCATCAAAGCATGCCAACTAGAAGAGGTAAGAAACTATGT | 32156 (32159) |
| MT (template for PCR) | CCTGGCACCATTAAAGAAAATATCATCTTTGGTGTTTCCTATGATGAATATGGATACAGAAGCGTCATCAAAGCATGCCAACTAGAAGAGGTAAGAAACTATGT | 32172 (32175) |

For structures of X_1_–X_23_ see Table S1; all dyes structures are in Figure 2 (main paper) and FAM-C6 is the same structure as FAM but with a C6 linker instead of C3. P = 3′-propanol PCR blocker; the position of the mutation to be detected is underlined. Unless otherwise stated oligonucleotides are DNA.

### Room Temperature Fluorescence Analysis

Fluorescence studies were performed on a Perkin Elmer LS50B luminescence spectrometer fitted with a Perkin Elmer PTP-1 Peltier temperature controller. FLWinlab TempScan software was used with settings of 600 nm min^-1^ scan speed. Optimum excitation wavelength, scan range wavelengths and excitation/emission slit width were used for each individual sample.

**Table S5.** Conditions for fluorescence scan analysis

|  | **Start (nm)** | **End (nm)** | **Excitation (nm)** | **Excitation Slit** | **Emission Slit** | **Emission Read (nm)** |
| --- | --- | --- | --- | --- | --- | --- |
| **TO** | 515 | 750 | 510 | 6 | 7 | 530 |
| **TO/FAM** | 515 | 750 | 510 | 4 | 5 | 530 |
|  | 495 | 750 | 490 | 4 | 5 | 530 |
| **TO/HEX** | 515 | 750 | 510 | 4 | 5 | 553 |
|  | 540 | 750 | 536 | 4 | 5 | 553 |
| **TO/ROX** | 515 | 750 | 510 | 6 | 7 | 607 |
|  | 585 | 750 | 585 | 6 | 7 | 607 |
| **TO/ATTO647N** | 515 | 750 | 510 | 6 | 7 | 665 |
|  | 650 | 750 | 647 | 6 | 7 | 665 |
| **BO** | 460 | 750 | 453 | 6 | 7 | 479 |
| **BO/FAM** | 460 | 750 | 454 | 4 | 5 | 518 |
|  | 500 | 750 | 492 | 4 | 5 | 518 |
| **BO/HEX** | 460 | 750 | 455 | 4 | 5 | 553 |
|  | 545 | 750 | 540 | 4 | 5 | 553 |
| **BO/ROX** | 460 | 750 | 453 | 6 | 7 | 607 |
|  | 590 | 750 | 586 | 6 | 7 | 607 |
| **BO/ATTO647N** | 460 | 750 | 452 | 6 | 7 | 665 |
|  | 650 | 750 | 647 | 6 | 7 | 665 |

In cases where several samples were compared, the slit width and excitation wavelength settings were kept constant. When multiple dyes were compared excitation wavelength was taken as the absorption maximum as specified in the experimental section.

The samples were prepared in phosphate buffer; NaH_2_PO_4_, 10 mM, 200 mM NaCl at pH 7.4, with labelled oligonucleotide concentration of 0.3 µM (0.36 μM for target strand). The oligonucleotide duplexes were heated to 95 °C to denature for 5 min before being allowed to slowly cool to room temperature to anneal, then analysed in a 200 μL quartz cuvette (Hellma quartz ‘SUPRASIL QS’; 200 μL volume, 1 cm pathlength) with a collection angle of 90°. The final spectra are an average of three.


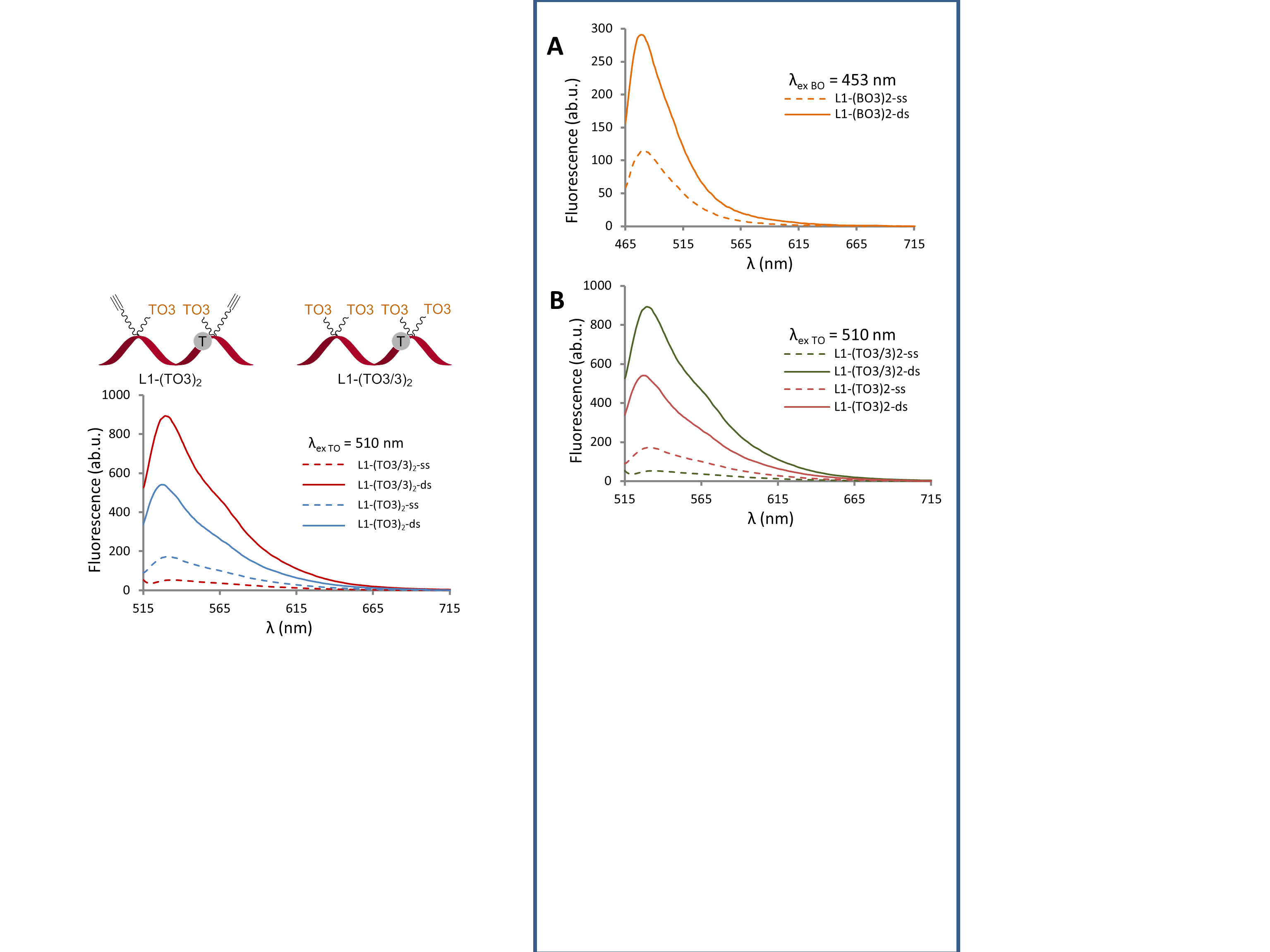


**Figure S2.** Multiple TO dyes give increased fluorescence enhancement on hybridisation to complementary DNA. Room temperature steady state emission of L1-(TO3)_2_ and L1-(TO3/3)_2_ in the single-stranded (ss) and double-stranded (ds) states. The probes are shown schematically (top). The T in the schematic highlights the position of the mutation to be detected relative to the positions of the fluorophores.


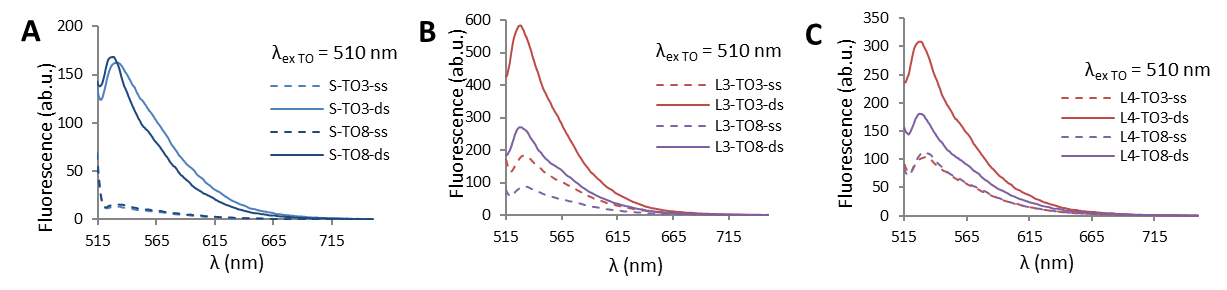


Figure S3. Room temperature steady state emission (λ_ex TO_ = 510 nm) of single TO-labelled oligonucleotides (S = short, L = long) in the single-stranded (ss) and double-stranded (ds) states.

**
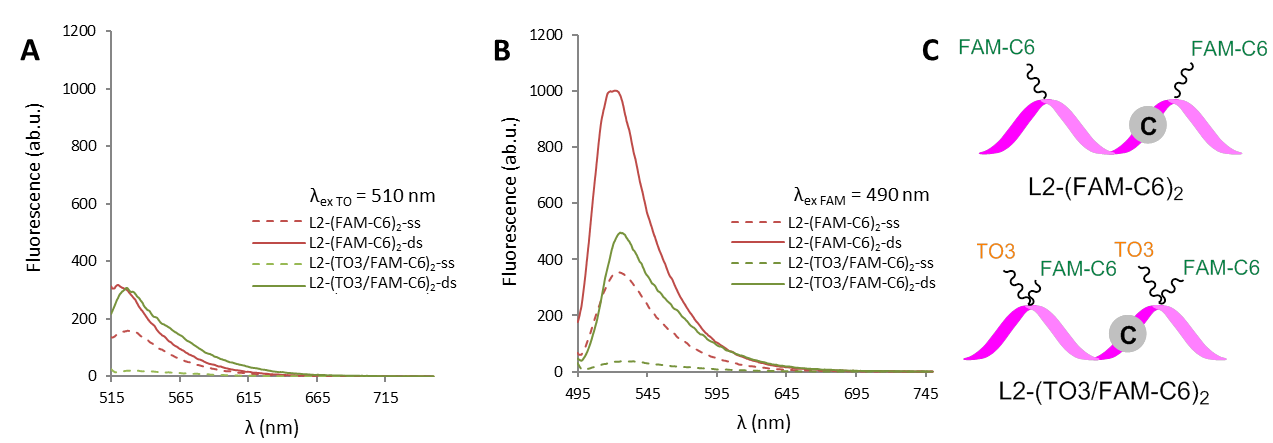
**

**Figure S4.** Comparison of room temperature steady state emission of the 22-mer probe L2-(TO3/FAM‑C6)_2_ and its control non-intercalative probe L2-(FAM-C6)_2_ in the single-stranded (ss) and double-stranded (ds) states (FAM-C6 is same structure as FAM in Figure 2, but with a C6 linker instead of C3). A: λ_ex_ = 510 nm (the excitation maximum of TO3); B: λ_ex_ = 490 nm (the excitation maximum of FAM-C6); C: schematic of probes, the nucleobase opposite the site of mutation to be detected is highlighted.


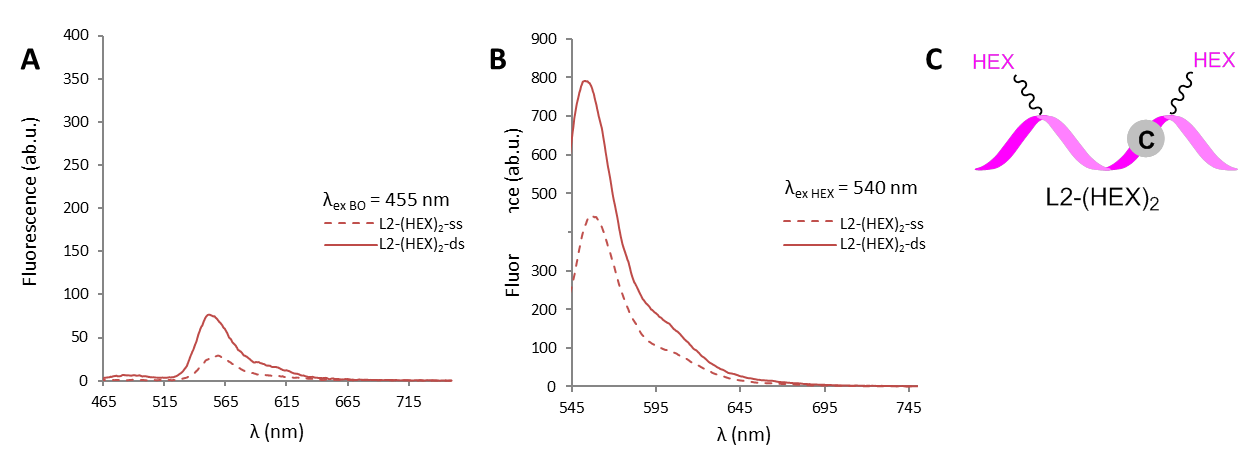


**Figure S5.** Room temperature steady state emission of the 22-mer probe L2-(HEX)_2_ in the single-stranded (ss) and double-stranded (ds) states. A: λ_ex_ = 455 nm (the excitation maximum of BO3); B: λ_ex_ = 536 nm (the excitation maximum of HEX); C: schematic of probes, the nucleobase opposite the site of mutation to be detected is highlighted.


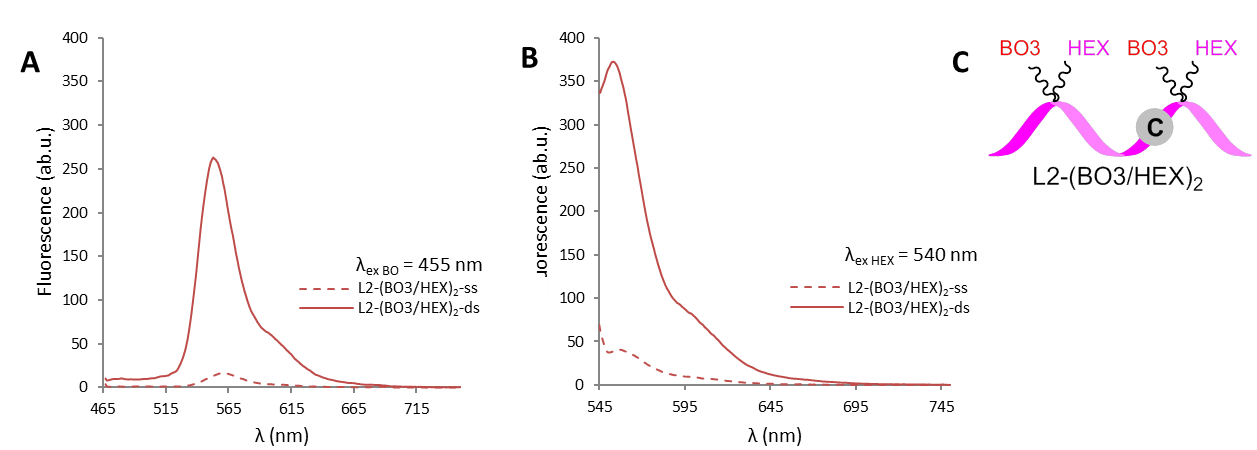


**Figure S6.** Room temperature steady state emission of the 22-mer probe L2-(BO3/HEX)_2_ in the single‑stranded (ss) and double-stranded (ds) states. A: λ_ex_ = 455 nm (the excitation maximum of BO3); B: λ_ex_ = 540 nm (the excitation maximum of HEX); C: schematic of probe, the nucleobase opposite the site of mutation to be detected is highlighted.


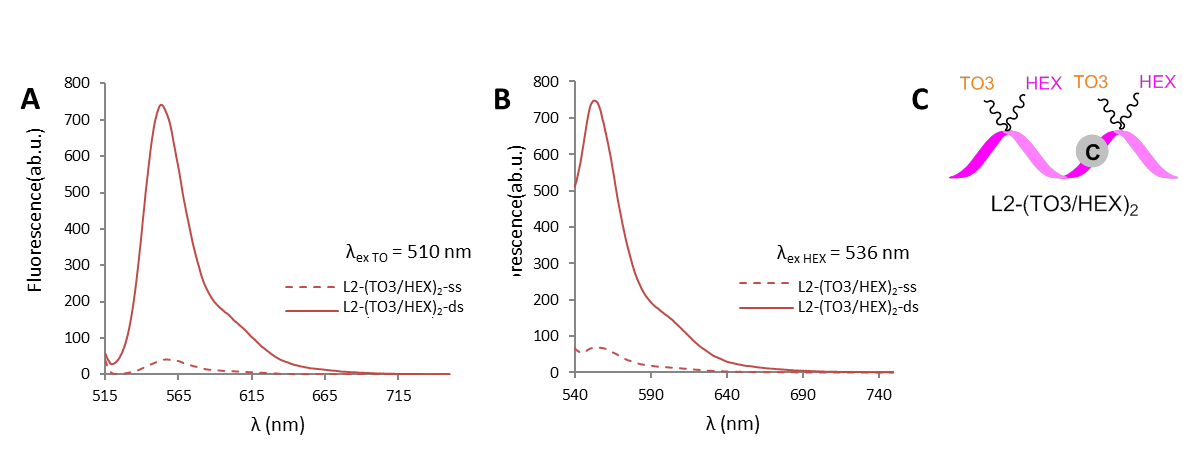


**Figure S7.** Room temperature steady state emission of the 22-mer probe L2-(TO3/HEX)_2_ in the single‑stranded (ss) and double-stranded (ds) states. A: λ_ex_ = 510 nm (the excitation maximum of TO3); B: λ_ex_ = 536 nm (the excitation maximum of HEX); C: schematic of probes, the nucleobase opposite the site of mutation to be detected is highlighted.


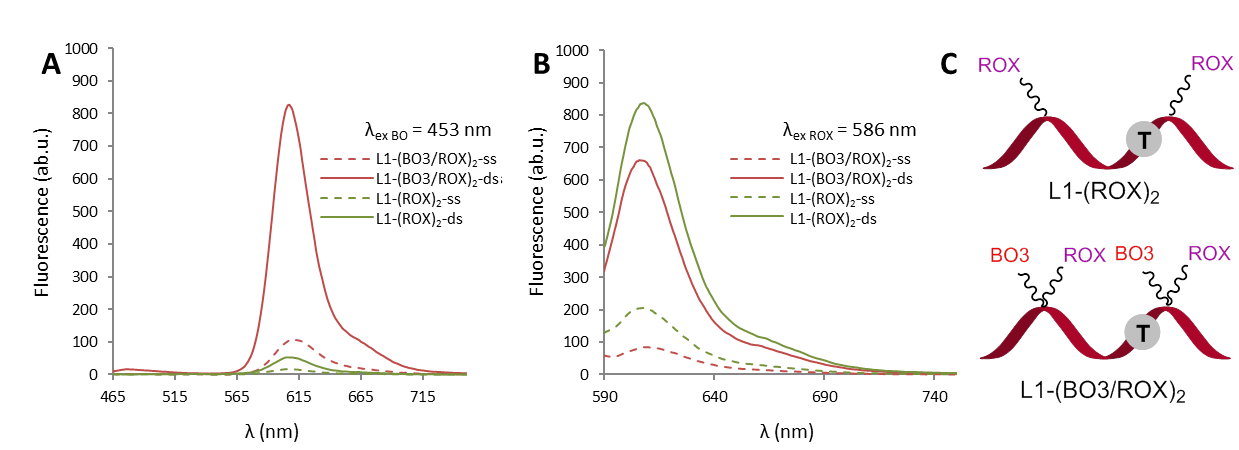


**Figure S8.** Comparison of room temperature steady state emission of the 22-mer probe L1-(BO3/ROX)_2_ and its control non-intercalative probe L1-(ROX)_2_ in the single-stranded (ss) and double-stranded (ds) states. A: λ_ex_ = 453 nm (the excitation maximum of BO3); B: λ_ex_ = 586 nm (the excitation maximum of ROX); C: schematic of probes, the nucleobase opposite the site of mutation to be detected is highlighted.

###
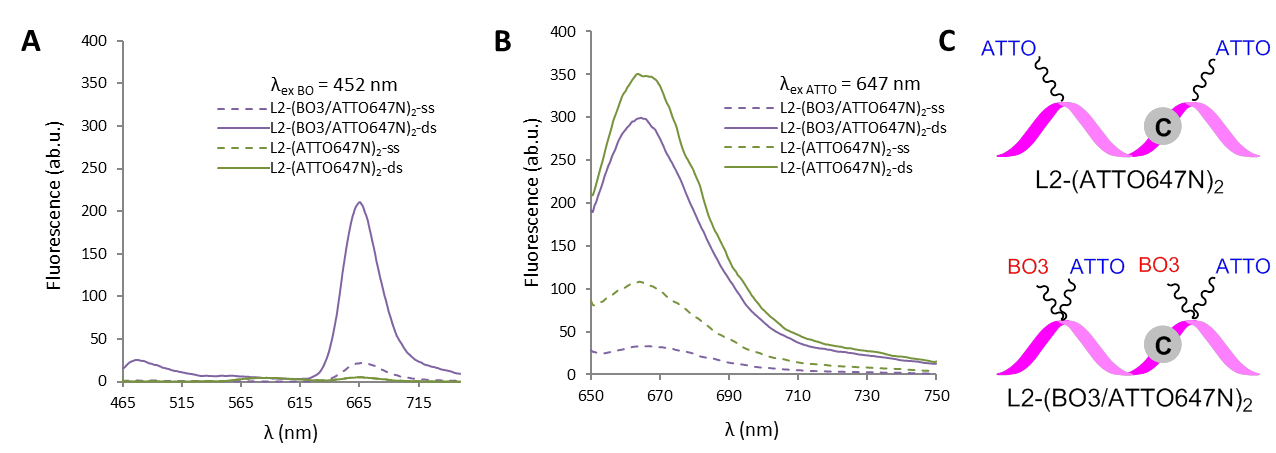


**Figure S9.** Comparison of room temperature steady state emission of the 22-mer probe L2-(BO/ATTO647N)_2_ and its control non-intercalative probe L2-(ATTO647N)_2_ in the single-stranded (ss) and double-stranded (ds) states. A: λ_ex_ = 452 nm (the excitation maximum of BO3); B: λ_ex_ = 647 nm (the excitation maximum of ATTO647N); C: schematic of probes, the nucleobase opposite the site of mutation to be detected is highlighted.

### Asymmetric PCR using BioRad CFX96 and Roche LightCycler Real-Time PCR Instruments

All PCR reaction samples were prepared under sterilised conditions in a LabCaire PCR workstation. All oligonucleotide sequences are in Table S4

Sample conditions:

Template: 1 ng per sample (WT or MT, Table S4)

Forward primer: 0.05 μM

Reverse primer: 0.5 μM

Probe (WT or MT): 0.5 μM L1-(TO3/ROX)_2_, L1-(BO3/ROX)_2_,
L2-(TO3/ATTO647N)_2_, L2-(BO3/ATTO647N)_2_, L1-(BO3/FAM)_2_, L1-(TO3/FAM)_2_, L2-(TO3/FAM-C6)_2_, L2-(BO3/HEX)_2_ or L2-(TO3/HEX)_2_.

### Buffer and DNA polymerases:

### GoTaq (1 unit) or KOD XL DNA (0.5 unit) Polymerases were used

5 × Colorless GoTaq reaction buffer (4 µL) or 10 × KOD XL DNA Polymerase buffer (2 µL).

500 μM each dNTP

Total volume: 20 μL

A negative control sample with no template was prepared for each PCR reaction.

Amplification was performed on a real-time PCR instruments (Bio-Rad CFX96 or Roche LightCycler) using the following procedure: an initial denaturing at 95 °C for 2 min, followed by 20–30 cycles of denaturing at 95 °C for 30 s, primer annealing at 53 °C for 30 s, and extension at 72 °C for 30 s. Further extension was carried out at 72 °C for 5 min. This was followed by melting experiments involving heating the amplification mixture to 95 °C for 30 s, cooling to 30 °C, then increasing the temperature at 1 °C s^-1^ to 95 °C and holding at each temperature for 5 s.

### 1- Asymmetric PCR using BioRad CFX96 Real-Time PCR Instrument

Reactions were undertaken using a BioRad CFX96 Real-Time PCR Instrument, with CFX Manager software (BioRad), monitoring in the following channels: FAM channel (excitation range 450–490 nm, detector range 510–530 nm), HEX channel (excitation range 515–535 nm, detector range 560–580 nm), ROX channel (excitation range 560–590 nm, detector range 610–650 nm) and Cy5 channel (excitation range 620–650 nm, detector range 675–690 nm). All PCR reactions were prepared under sterilised conditions in a LabCaire PCR workstation.

Reactions were run in 0.2 mL low-profile white 8-tube strips with optically clear lids (BioRad). Sample preparation and thermal protocols for each PCR probe and polymerase are detailed above.


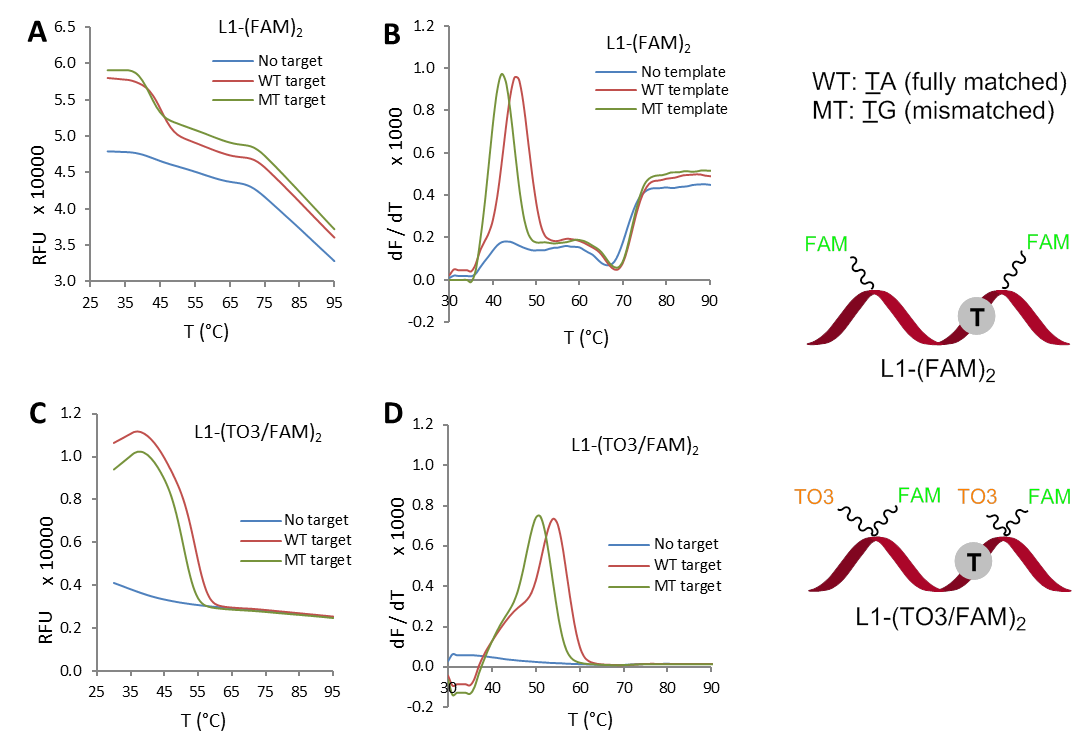


**Figure S10.** Fluorescence melting curves (A, C) and derivatives (B, D) of post-amplification fluorescence melts using the wild-type probe L1-(TO3/FAM)_2_ (C, D) and the corresponding control non-intercalative probe L1-(FAM)_2_ (A, B). All output was monitored in the FAM channel of the CFX96 real-time PCR instrument (excitation range 450–490 nm, detector range 510–530 nm). GoTaq DNA polymerase, 30 cycles and 1 ng of template oligonucleotides were used. L1-(TO3/FAM)_2_ is a wild-type probe that gives a fully matched duplex upon hybridization with wild-type template (WT), and forms a T:G‑mismatched duplex when paired with mutant template (MT). The T in the schematic diagram indicates the position of the nucleobase opposite the site of the point mutation to be detected.


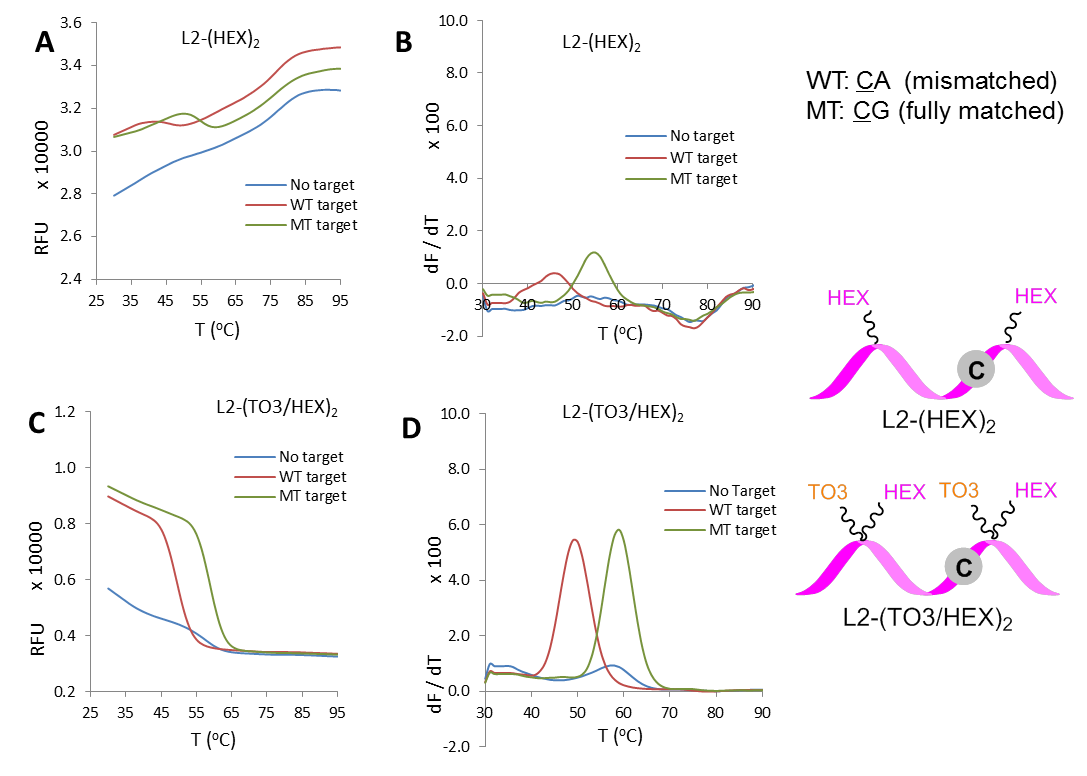


**Figure S11.** Fluorescence melting curves (A, C) and derivatives (B, D) of post-amplification fluorescence melts using the mutant probe L2-(TO3/HEX)_2_ (C, D) and its control non-intercalative probe L2-(HEX)_2_ (A, B). All output was monitored in the HEX channel of the CFX96 real‑time PCR instrument (excitation range 515–535 nm, detector range 560–580 nm). KOD XL DNA polymerase, 20 cycles and 1 ng of template oligonucleotides were used. L2-(TO3/HEX)_2_ is a mutant probe that gives a C:A‑mismatched duplex upon hybridization with wild-type template (WT), and forms a fully matched duplex when paired with mutant template (MT). The C in the schematic indicates the position of this nucleobase opposite the site of the point mutation to be detected.


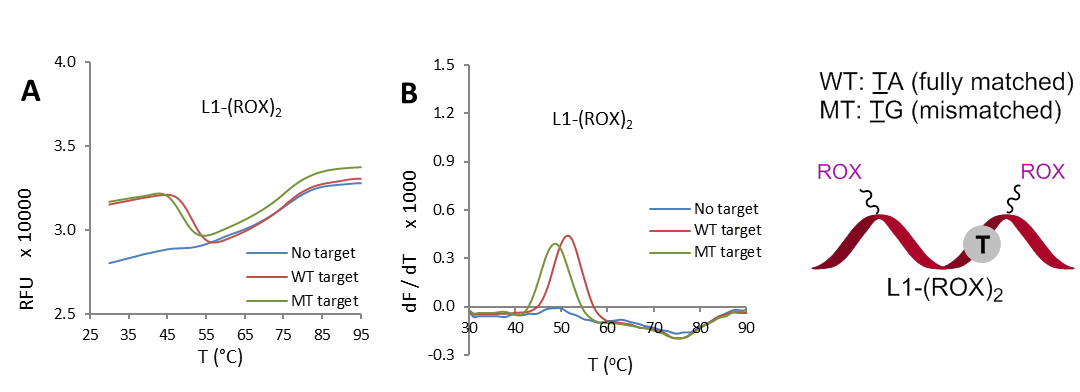


**Figure S12.** Fluorescence melting curves (A) and derivatives (B) of post-amplification fluorescence melts using the wild-type non-intercalative probe L1-(ROX)_2_. All output was monitored in the ROX channel of the CFX96 real-time PCR instrument (excitation range 560–590 nm, detector range 610–650 nm). KOD XL DNA polymerase, 20 cycles and 1 ng of template oligonucleotides were used. L1-(ROX)_2_ is a wild-type probe that gives a fully matched duplex upon hybridization with wild‑type template (WT), and forms a T:G-mismatched duplex when paired with mutant template (MT). The T in the schematic indicates the position of this nucleobase opposite the site of the point mutation to be detected.


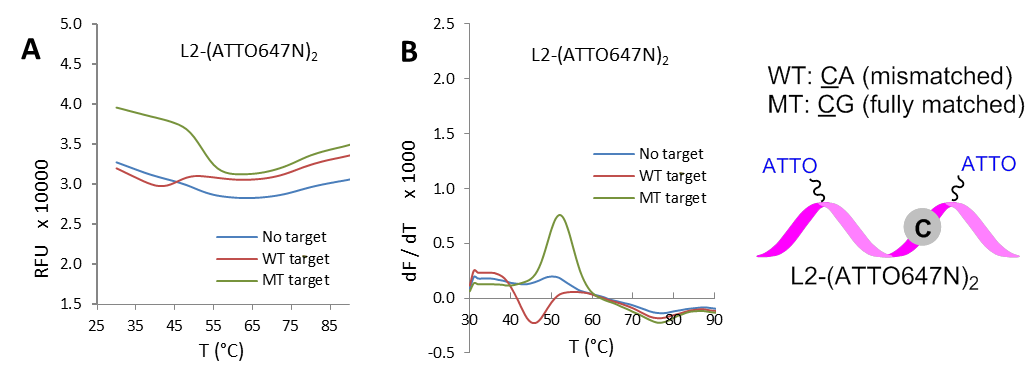


**Figure S13.** Fluorescence melting curves (A) and derivatives (B) of post-amplification fluorescence melts using the mutant non-intercalative probe L2-(ATTO647N)_2_. All output was monitored in the Cy5 channel of the CFX96 real-time PCR instrument (excitation range 620–650 nm, detector range 675–690 nm). KOD XL DNA polymerase, 20 cycles and 1 ng of template were used. L2-(ATTO647N)_2_ is a mutant probe that gives a C:A‑mismatched duplex upon hybridization with the wild‑type template (WT), and forms a fully matched duplex when paired with the mutant template (MT). The C in the schematic indicates the position of this nucleobase opposite the site of the point mutation to be detected.


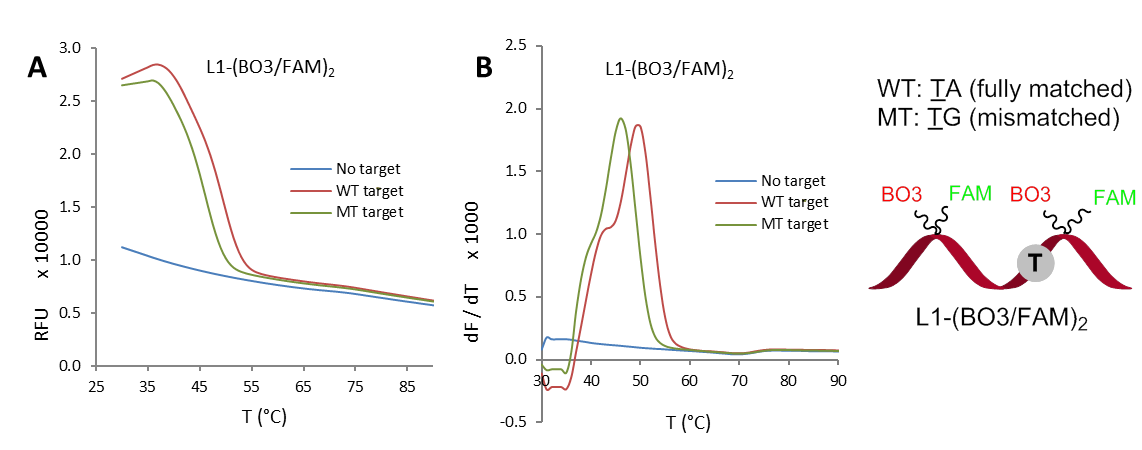


**Figure S14.** Fluorescence melting curves (A) and derivatives (B) of post-amplification fluorescence melts using the wild-type probe L1-(BO3/FAM)_2_. All output was monitored in the FAM channel of the CFX96 real-time PCR instrument (excitation range 450–490 nm, detector range 510–530 nm). GoTaq DNA polymerase, 30 cycles and 1 ng of target oligonucleotides were used. L1-(BO3/FAM)_2_ is a wild‑type probe that gives a fully matched duplex upon hybridization with the wild‑type template (WT), and forms a T:G-mismatched duplex when paired with the mutant template (MT). The T in the schematic indicates the position of this nucleobase opposite the site of the point mutation to be detected.


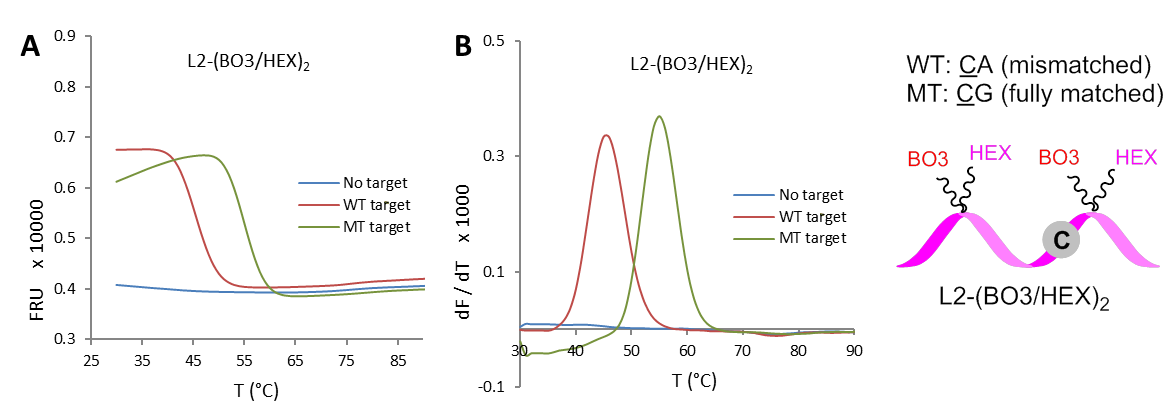


**Figure S15.** Fluorescence melting curves (A) and derivatives (B) of post-amplification fluorescence melts using the mutant probe L2-(BO3/HEX)_2_. All output was monitored in the HEX channel of the CFX96 real-time PCR instrument (excitation range 515–535 nm, detector range 560–580 nm). KOD XL DNA polymerase, 20 cycles and 1 ng of target oligonucleotides were used. L2-(BO3/HEX)_2_ is a mutant probe that gives a C:A‑mismatched duplex upon hybridization with the wild‑type template (WT), and forms a fully matched duplex when paired with the mutant template (MT). The C in the schematic indicates the position of this nucleobase opposite the site of the point mutation to be detected.


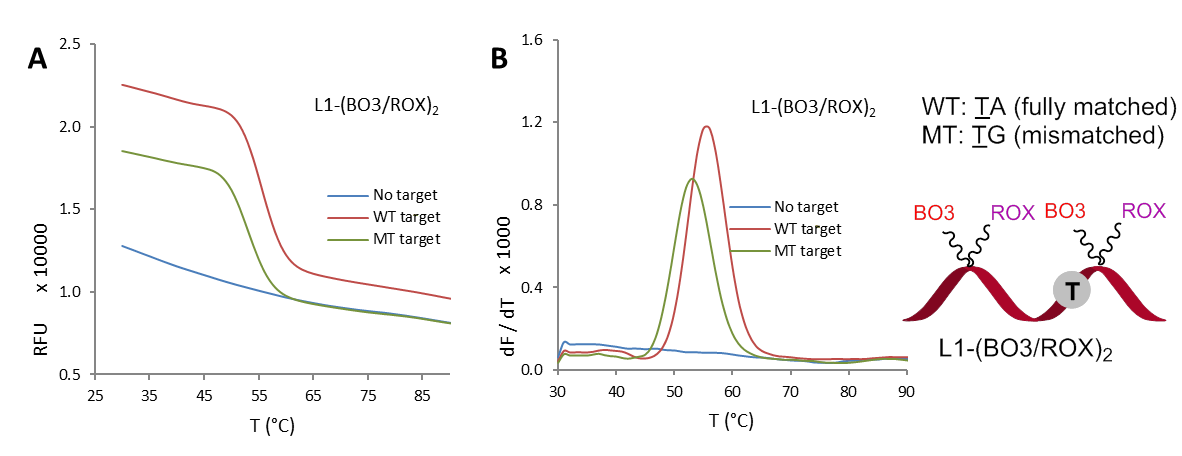


**Figure S16.** Fluorescence melting curves (A) and derivatives (B) of post-amplification fluorescence melts using the wild-type probe L1-(BO3/ROX)_2_. All output was monitored in the ROX channel of the CFX96 real-time PCR instrument (excitation range 560–590 nm, detector range 610–650 nm). KOD XL DNA polymerase, 20 cycles and 1 ng of template oligonucleotides were used. L1-(BO3/ROX)_2_ is a wild‑type probe that gives a fully matched duplex upon hybridization with the wild‑type template (WT), and forms a T:G-mismatched duplex when paired with the mutant template (MT). The T in the schematic indicates the position of the nucleobase opposite the site of the point mutation to be detected.


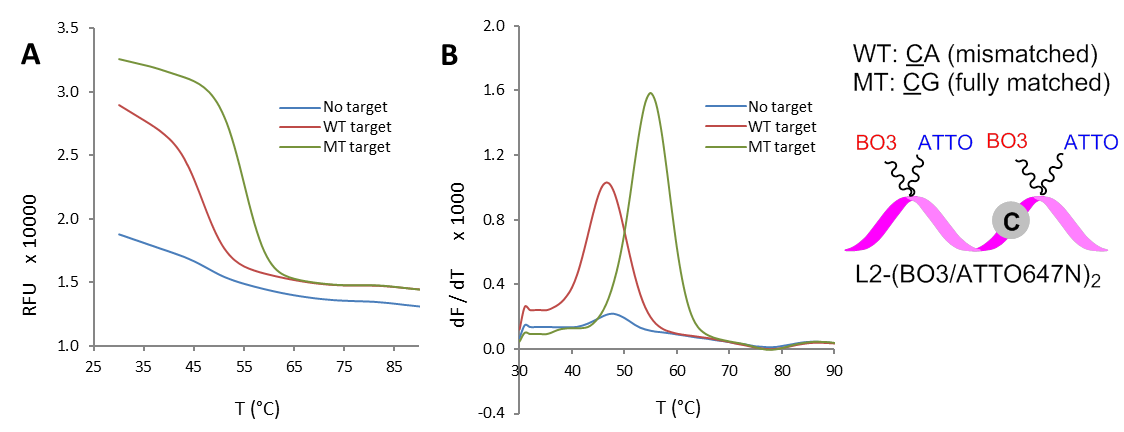


**Figure S17.** Fluorescence melting curves (A) and derivatives (B) of post-amplification fluorescence melts using the mutant probe L2-(BO3/ATTO647N)_2_. All output was monitored in the Cy5 channel of the CFX96 real-time PCR instrument (excitation range 620–650 nm, detector range 675–690 nm). KOD XL DNA polymerase, 20 cycles and 1 ng of template oligonucleotides were used. Note: L2-(BO3/ATTO647N)_2_ is a mutant probe that gives a C:A‑mismatched duplex upon hybridization with the wild-type template (WT), and forms a fully matched duplex when paired with the mutant template (MT). The C in the schematic indicates the position of the nucleobase opposite the site of the point mutation to be detected.

### 2- Asymmetric PCR using Roche LightCycler 1.5 instrument

Samples were prepared in LightCycler glass capillaries (20 µL volume) and Roche LightCycler Software Version 3.5 was used. The LightCycler has one excitation source (488 nm) and three channels for recording fluorescence emission at 530 nm (F1), 640 nm (F2) and 705 (F3). The melting profiles for up to 32 samples can be recorded simultaneously.

For asymmetric PCR reactions (sample preparation, see page S37), an initial denaturing at 95 °C for 2 min was followed by 30 cycles of denaturing at 95 °C for 30 s, primer annealing at 53 °C for 30 s, and extension at 72 °C for 30 s. Further extension was carried out at 72 °C for 5 min. This was followed by fluorescence melting analysis.

Fluorescence melting: the samples (0.5 µM, 20 µL) were first denatured by heating to 95 °C at a rate of 20.0 °C s^–1^. The samples were then maintained at 95 °C for 2 min before annealing by cooling to 30 °C at 0.1 °C s^–1^. They were held at 30 °C for a further 5 min and then melted by heating to 95 °C at 0.1 °C s^–1^. The fluorescence melting data were converted to the first derivatives to give the melting peaks -d(F)/dT, where F is fluorescence and T is temperature in °C. Recordings were taken during the melting and annealing steps.


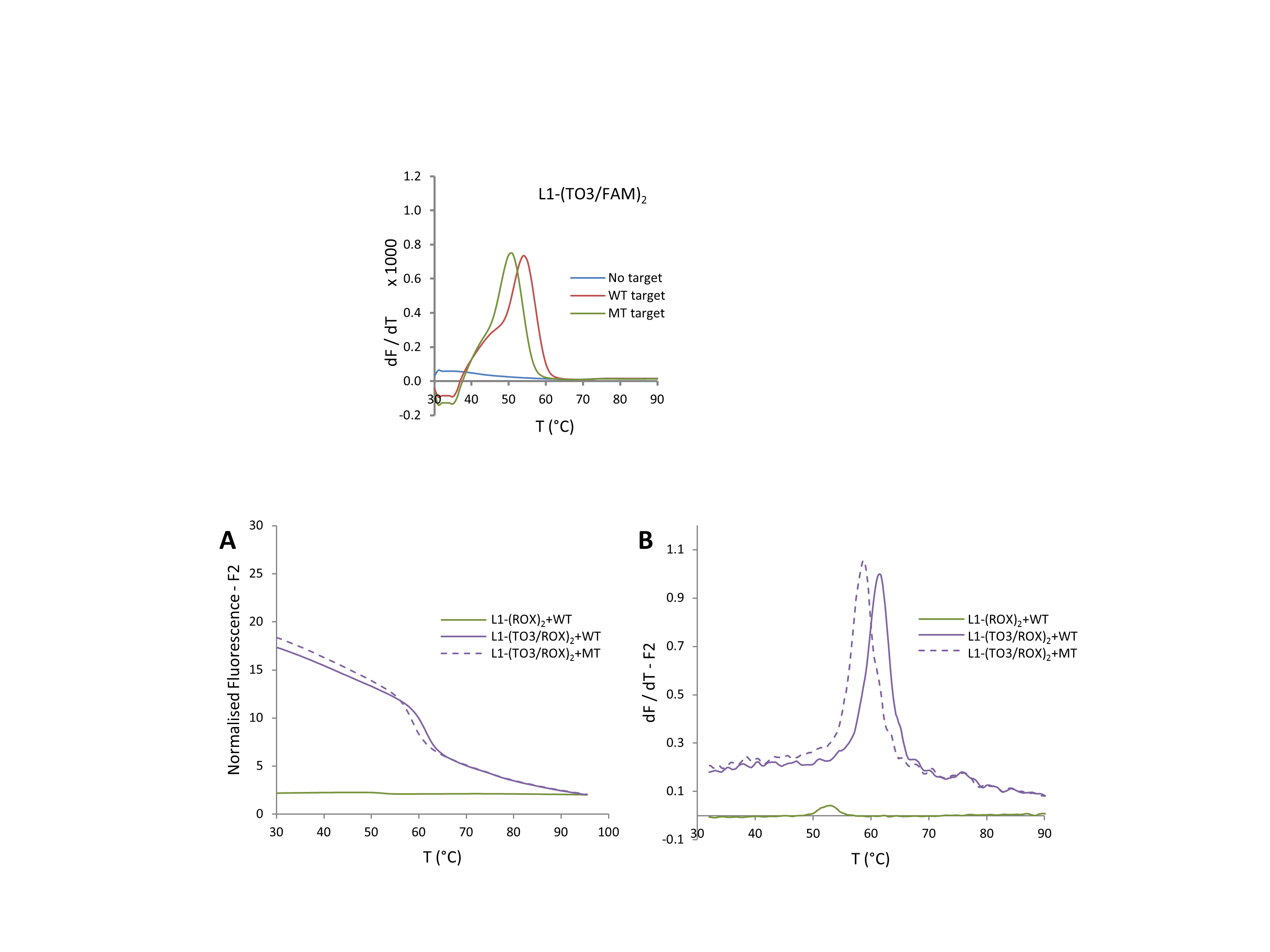


**Figure S18.** Fluorescence melting curves (A) and derivatives (B) of post-amplification fluorescence melts using the wild-type probe L1-(TO3/ROX)_2_ and its non-intercalative probe L1-(ROX)_2_. All output was monitored in the F2 channel (640 nm) of the Roche LightCycler instrument. KOD XL DNA polymerase, 30 cycles of PCR and 1 ng of target oligonucleotides were used. L1-(TO3/ROX)_2_ and L1-(ROX)_2_ are wild type probes that give T:G‑mismatched duplexes upon hybridization with the mutant template (MT), and form fully matched duplexes when paired with the wild type template (WT).

**References**

1. Gottlieb, H.E., Kotlyar, V. and Nudelman, A. (1997) NMR chemical shifts of common laboratory solvents as trace impurities. *J. Org. Chem.*, **62**, 7512-7515.

2. Kabatc, J. and Jurek, K. (2015) New two- and three-cationic polymethine dyes. Synthesis, properties and application. *Dyes Pigment.*, **112**, 24-33.

3. Lartia, R. and Asseline, U. (2006) New cyanine-oligonucleotide conjugates: Relationships between chemical structures and properties. *Chem.-Eur. J.*, **12**, 2270-2281.

4. Holzhauser, C., Rubner, M.M. and Wagenknecht, H.A. (2013) Energy-transfer-based wavelength-shifting DNA probes with "clickable" cyanine dyes. *Photochem. Photobiol. Sci.*, **12**, 722-724.

5. Hansen, A.S., Thalhammer, A., El-Sagheer, A.H., Brown, T. and Schofield, C.J. (2011) Improved synthesis of 5-hydroxymethyl-2 '-deoxycytidine phosphoramidite using a 2 '-deoxyuridine to 2 '-deoxycytidine conversion without temporary protecting groups. *Bioorg. Med. Chem. Lett.*, **21**, 1181-1184.

6. Sirivolu, V.R., Chittepu, P. and Seela, F. (2008) DNA with Branched Internal Side Chains: Synthesis of 5-Tripropargylamine-dU and Conjugation by an Azide-Alkyne Double Click Reaction. *Chembiochem*, **9**, 2305-2316.

7. Schneider, S.K., Roembke, P., Julius, G.R., Raubenheimer, H.G. and Herrmann, W.A. (2006) Pyridin-, quinolin- and acridinylidene palladium carbene complexes as highly efficient C-C coupling catalysts. *Advanced Synthesis & Catalysis*, **348**, 1862-1873.
